# Biocompatible Phosphorescent O_2_ Sensors Based on Ir(III) Complexes for In Vivo Hypoxia Imaging

**DOI:** 10.3390/bios13070680

**Published:** 2023-06-26

**Authors:** Mozhgan Samandarsangari, Daria O. Kozina, Victor V. Sokolov, Anastasia D. Komarova, Marina V. Shirmanova, Ilya S. Kritchenkov, Sergey P. Tunik

**Affiliations:** 1 Institute of Chemistry, St. Petersburg State University, Universitetskaya Embankment 7-9, 199034 St. Petersburg, Russia; m.samandarsangari@spbu.ru (M.S.); st055671@student.spbu.ru (D.O.K.); v.sokolov@spbu.ru (V.V.S.); 2 Institute of Experimental Oncology and Biomedical Technologies, Privolzhskiy Research Medical University, Minin and Pozharsky Sq. 10/1, 603005 Nizhny Novgorod, Russia; komarova_a@pimunn.net (A.D.K.); shirmanovam@mail.ru (M.V.S.); 3 Institute of Biology and Biomedicine, Lobachevsky State University of Nizhny Novgorod, Gagarina Av., 23, 603950 Nizhny Novgorod, Russia

**Keywords:** oxygen sensing, iridium complexes, phosphorescence, hypoxia, bioimaging, phosphorescence lifetime imaging

## Abstract

In this work, we obtained three new phosphorescent iridium complexes (**Ir1**–**Ir3**) of general stoichiometry [Ir(N^C)_2_(N^N)]Cl decorated with oligo(ethylene glycol) fragments to make them water-soluble and biocompatible, as well as to protect them from aggregation with biomolecules such as albumin. The major photophysical characteristics of these phosphorescent complexes are determined by the nature of two cyclometallating ligands (N^C) based on 2-pyridine-benzothiophene, since quantum chemical calculations revealed that the electronic transitions responsible for the excitation and emission are localized mainly at these fragments. However, the use of various diimine ligands (N^N) proved to affect the quantum yield of phosphorescence and allowed for changing the complexes’ sensitivity to oxygen, due to the variations in the steric accessibility of the chromophore center for O_2_ molecules. It was also found that the N^N ligands made it possible to tune the biocompatibility of the resulting compounds. The wavelengths of the **Ir1**–**Ir3** emission maxima fell in the range of 630–650 nm, the quantum yields reached 17% (**Ir1**) in a deaerated solution, and sensitivity to molecular oxygen, estimated as the ratio of emission lifetime in deaerated and aerated water solutions, displayed the highest value, 8.2, for **Ir1**. The obtained complexes featured low toxicity, good water solubility and the absence of a significant effect of biological environment components on the parameters of their emission. Of the studied compounds, **Ir1** and **Ir2** were chosen for in vitro and in vivo biological experiments to estimate oxygen concentration in cell lines and tumors. These sensors have demonstrated their effectiveness for mapping the distribution of oxygen and for monitoring hypoxia in the biological objects studied.

## 1. Introduction

The development and application of transition metal phosphorescent complexes, such as non-invasive molecular oxygen sensors, is a topical area of research. The possibility of using such complexes in biomedical experiments is particularly important, since tracking changes in O_2_ concentration is a fundamental issue in the studies of metabolic processes, as well as for diagnosing various pathologies and evaluating the efficiency of the therapy used [1,2,3,4]. The sensory response of phosphorescent complexes to the presence of molecular oxygen is based on the effective energy transfer from the excited triplet state of phosphors to the ground triplet state of O_2_ molecules to give phosphorescence quenching, accompanied by a decrease in emission intensity and in the lifetime of the excited state [5,6,7].

In early studies, phosphorescence intensity was actively used as an analytical signal to determine oxygen concentration. In the ratiometric approach, the sensor response was quantified by comparison of the phosphorescence intensity with that of a certain oxygen-independent external or internal standard; fluorescence emission was commonly used as the latter. However, this approach makes analytical systems more complicated (at least two emitters have to be used in the measurements) and suffers from the dependence of the results on the optical properties of the samples under study, as well as the possible influence of different factors, other than oxygen concentration, on the luminescence of the reference emitter (pH, temperature, viscosity, etc.). The lifetime response of oxygen concentration variations is free from the above drawbacks, does not depend on the sensor concentration that makes phosphorescence lifetime measurements more reliable and results in a wide use of phosphoresce lifetime imaging (PLIM) in different analytical and biomedical applications, including oxygen sensing [7,8,9,10].

For the successful application of phosphorescent oxygen sensors in biology, they should be soluble in physiological (aqueous) media, demonstrate low toxicity and high stability and exhibit good photophysical characteristics (high quantum yield, sensitivity to the presence of molecular oxygen, emission and excitation in a required wavelength range). It is also worth noting that, in order to verify the practicable applicability of oxygen sensors in biomedical experiments, it is necessary to test them in various model biological media, since different factors, such as variations in pH values, temperature, salinity, viscosity, ions and the presence of biomacromolecules (primarily albumin, which has in its structure so-called “hydrophobic pockets”), can significantly affect the photophysical characteristics of the sensor.

Among the most effective phosphorescent oxygen sensors which are also commercially available, it is worth mentioning those obtained in the research groups of Sergei Vinogradov (University of Pennsylvania) [11,12,13,14,15,16,17,18,19,20] and Dmitri Papkovsky (University College Cork) [21,22,23,24,25,26,27,28,29,30,31,32,33,34,35]. These sensors are based on Pt and Pd porphyrin chromophores, which are highly sensitive to oxygen and also protected from the external effects of the biological environment by either being encapsulated within dendrimer layers and subsequently modified by attaching large, linear poly(ethylene glycol) (PEG) chains [11,12,13,14,15,16,17,18,19,20] or by embedding the chromophore into hydrophilic nanoparticles [21,22,23,24,25,26,27,28,29,30,31,32,33,34,35]. However, despite their significant advantages, these sensors are used predominantly to measure the oxygen concentration in the extracellular environment due to the impenetrability of the cell membrane and their instability in an intracellular environment. Cell penetration can be increased by chromophore functionalization with relatively small glucose moiety [36], but it has been shown [37] that the obtained compounds display partial aggregation in aqueous solutions that is undesirable for biological applications. Platinum porphyrins’ conjugation with peptides [38] made it possible to design a cell-penetrating oxygen sensor, but this was based on rather complicated chemistry. It is also worth noting that porphyrin emitters also have rather high lifetimes of their excited states (from tens to hundreds of microseconds) that considerably lengthen the time of information acquisition compared with some other phosphorescent compounds, e.g., iridium complexes, which display essentially shorter lifetimes from hundreds of nanoseconds to a few microseconds.

Exploration of Ir(III) orthometallated complexes as oxygen sensors has a rather long and rich history, beginning with a key publication [39] demonstrating the applicability of this type of emitter for the detection of hypoxia in biomedical research. Later (see a mini-review [40] and recent review [41] for a complete list of references), in the works of Dr. S. Tobita et al. and several other research teams [36,42,43,44,45,46,47], it has been demonstrated that Ir(III) molecular emitters can be successfully applied in in vitro and in vivo biomedical studies to qualitatively detect hypoxia in the tumor microenvironment [45,48] and to measure oxygen concentration in cell cultures by using a ratiometric approach [49] or time-resolved techniques, such as PLIM [50,51]. Moreover, in some cases, directed subcellular localization is possible; for example, in mitochondria [44,52], which makes it possible to track (especially with simultaneous FLIM imaging of the NADH and other coenzymes) metabolic processes and their variations with changes in nutritional conditions, oxygenation, etc. Significantly shorter values of the lifetimes of iridium complexes compared with the platinum and palladium porphyrins (by one or two orders of magnitude) allow for much faster signal accumulation in PLIM experiments, making it possible to increase the acquisition rate and/or real-time image resolution. Nevertheless, it must be mentioned that the iridium chromophores display lower brightness compared with the above-mentioned Pt and Pd porphyrins, which is determined not by their quantum yields (which are quite comparable), but by the lower extinction coefficient of these samples. Nevertheless, the versatility of the photophysical characteristics of iridium complexes achieved by variations in the donor properties of the ligands and simpler synthetic methods open the way for their further modification and vectorization, making Ir(III) complexes the compounds of choice for the preparation of oxygen sensors for particular biological studies.

As for many other phosphorescent transition metal complexes, their hydrophobicity and insolubility in aqueous media remain issues to be solved. One of the useful approaches consists of chromophore conjugation with synthetic [45] or natural [53,54] water-soluble polymers. Recently, we have designed and synthesized a number of oxygen sensors based on iridium complexes [52,55,56,57,58,59,60,61,62,63,64,65], which exhibit good sensitivity to molecular oxygen. The chromophores in these compounds are shielded from interaction with the bio-environment by relatively short oligo(ethylene glycol) tails, which makes intracellular internalization possible and to simultaneously impart water solubility and increase the biocompatibility of these molecules.

In this article, we present the synthesis, characterization and photophysical study of three novel phosphorescent [Ir(N^C)_2_(N^N)]Cl complexes (**Ir1**–**Ir3**) containing the same N^C metallated and different N^N diimine ligands. The oxygen-sensing properties of the most effective emitters (**Ir1** and **Ir2**) were investigated in model physiological media and in living cells, as well as through in vivo experiments in a mice tumor model by using time-resolved phosphorescent lifetime imaging.

## 2. Materials and Methods

NMR spectra (1D ^1^H, 2D ^1^H-^1^H COSY and NOESY) were recorded on a Bruker 400 MHz Avance (Billerica, MA, USA); chemical shift values were referenced to the solvent residual signals. Mass spectra were recorded on a Bruker maXis HRMS-ESI-QTOF in the ESI^+^ mode.

Reagents: methyl 6-(benzo[b]thiophen-2-yl)nicotinate [66], 2,5,8,12,15,18-hexaoxanonadecan-10-amine [67] and N^N1-N^N3 ligands [52,60,62] were obtained according to the published procedures. The synthesis of 6-(benzo[b]thiophen-2-yl)nicotinic acid, N^C ligand, [Ir_2_(N^C)_4_Cl_2_] dimer and the target complexes **Ir1**–**Ir3** are described in the Supplementary Information file. Other solvents and reagents were received form BLD Pharmatech (Shanghai, China), Merck (Darmstadt, Germany), Vekton (St. Petersburg, Russia) and used without additional purification.

Details of synthetic and separation procedures, photophysical experiments and computational studies, as well as data concerning the description of installations and methods for conducting biological experiments, are given in the Supplementary Materials.

## 3. Results and Discussion

### 3.1. Synthesis and Characterization

A derivative of pyridine–benzothiophene was chosen as a cyclometallating ligand, since it is known that iridium complexes based on it exhibit emissions in the red region of the spectrum [68,69,70], which can be useful for in vivo studies, since such luminescence will be within the transparency window of biological tissues (≥600 nm).

**Scheme 1:**
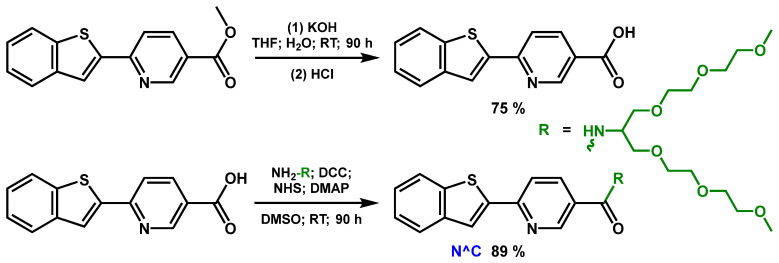
Synthetic scheme of the new ligand N^C.

To impart water solubility, biocompatibility and low toxicity to the target compounds, as well as to protect them from nonspecific interactions with biomolecules, we introduced short-branched oligo(ethylene glycol) substituents into the structure of the cyclometallating and diimine ligands. The scheme of the corresponding modification and obtaining a new N^C ligand is given below (Scheme 1), while the modification reactions of the corresponding N^N ligands are described in the previous literature [52,60,62].

**Scheme 2:**
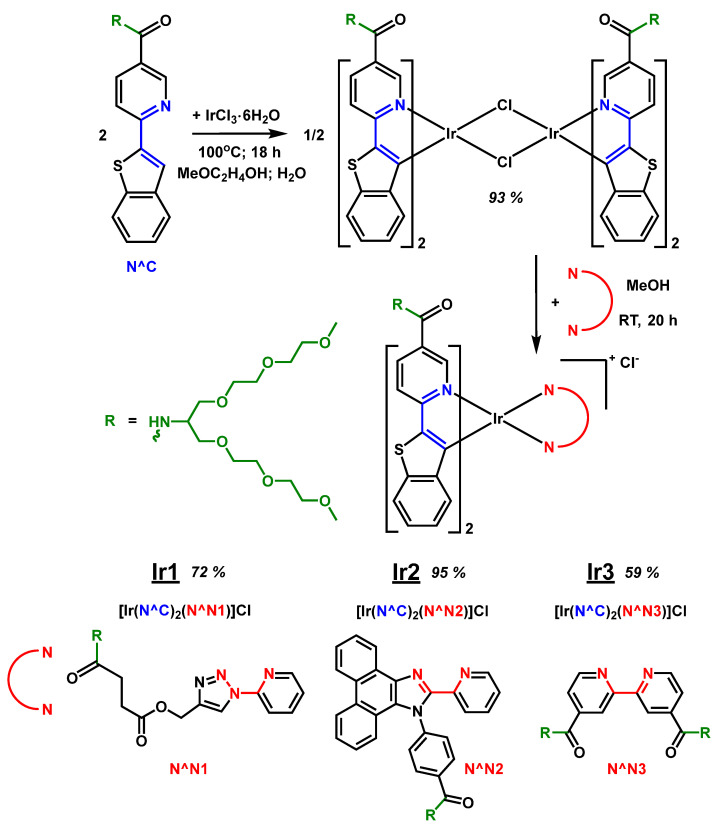
Scheme of synthesis of new Ir(III) dimer [Ir_2_(N^C)_4_Cl_2_] and new complexes **Ir1**–**Ir3**.

In the next stage of the synthesis (Scheme 2, upper part), upon the reaction of this cyclometallating ligand N^C and iridium(III) chloride, we isolated a new dimeric complex [Ir_2_(N^C)_4_Cl_2_], which we further used as a starting material in obtaining the target phosphorescent compounds, **Ir1**–**Ir3**. The synthesis of the target complexes (Scheme 2, bottom part) was carried out by replacing the labile chloride ligands in the [Ir_2_(N^C)_4_Cl_2_] dimer with diimine N^N chelates, with simultaneous dissociation of the dimer, to obtain the [Ir(N^C)_2_(N^N#)]Cl complexes in good preparative yields (59–95%), where the N^N# diimine ligands were also modified with oligo(ethylene glycol) residues.

The compounds obtained (ligands, iridium(III) dimer and the target iridium complexes) were comprehensively characterized by NMR spectroscopy and mass spectrometry. It should be noted that, due to the presence of a large number of oligo(ethylene glycol) substituents in the structure of the target complexes, we failed to obtain these compounds either in the form of high-quality single crystals, suitable for X-ray diffraction analysis, or as a polycrystalline material, suitable for elemental analysis of the CHNS content. Nevertheless, the number, multiplicity, location and integral intensities of ^1^H signals in 1D NMR spectra, as well as their cross-correlations in 2D ^1^H-^1^H COSY and NOESY NMR spectra, made it possible to reliably establish the structure and composition of these compounds (Figures S1–S17 in the Supplementary Materials). Additionally, these conclusions were confirmed by high-resolution ESI^+^ mass spectrometry; see Figures S3–S18. In the obtained mass spectra, the main signals corresponded to the molecular ions of these complexes, both in pure form and with the addition of H^+^ or Na^+^ cations. The isotopic distribution patterns were also in excellent agreement with those calculated for these particles.

**Figure 1:**
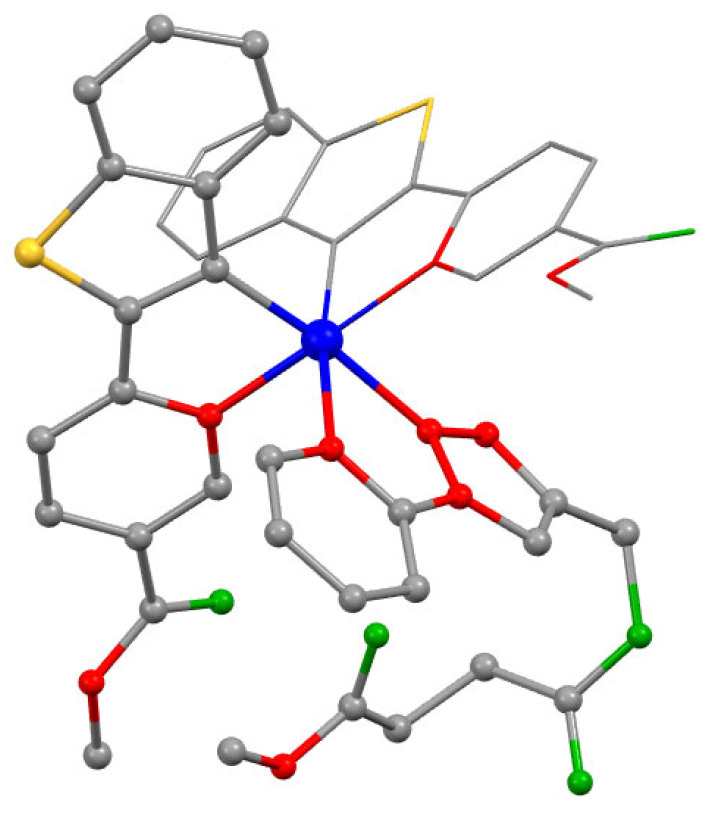
Optimized structure of the model **Ir1**–**0** complex (hydrogen atoms are omitted for clarity). The calculations have been simplified by substitution of OEG pendants in **Ir1** complex for methyl groups in **Ir1**–**0** structure. Atom colors: Ir—blue; S—yellow; O—green; N—red; C—gray.

We also carried out quantum chemical calculations, which included optimization of the ground state structure (as an example, the optimized structure of the **Ir1**–**0** complex is shown in Figure 1). Note that for the sake of simplicity in the optimization procedure, we used a model structural pattern, which differs from the structures of the **Ir1**–**Ir3** compounds in that oligo(ethylene glycol) groups were replaced by methyl substituents to reduce the calculation time. It should be noted that such substitution is reasonable, since neither oligo(ethylene glycol) nor methyl substituents noticeably affect the central core structure and photophysical characteristics, being remote from the chromophoric center and having a similar nature from the viewpoint of donor–acceptor properties. The optimized structures for the **Ir2** and **Ir3** complexes are shown in Figures S22 and S23 in the Supplementary Materials. The obtained characteristics of the optimized structures (ligand disposition in the coordination octahedron, bond lengths and angles) are not exceptional and fit the structures of closely analogous complexes well, synthesized and characterized previously [68,69,70]. The obtained structural parameters of the optimized patterns are summarized in Tables S9–S11; see Supplementary Materials. It is also important to note that the optimized structural motifs of **Ir1**–**Ir3** are in complete agreement with the data of proton NMR spectroscopy in terms of the ligand environment symmetry and intramolecular non-bonding proton contacts observed in the NOESY spectra, which additionally confirm the correctness of the complexes’ composition and structure determination by this method.

### 3.2. Photophysical Study

**Figure 2:**
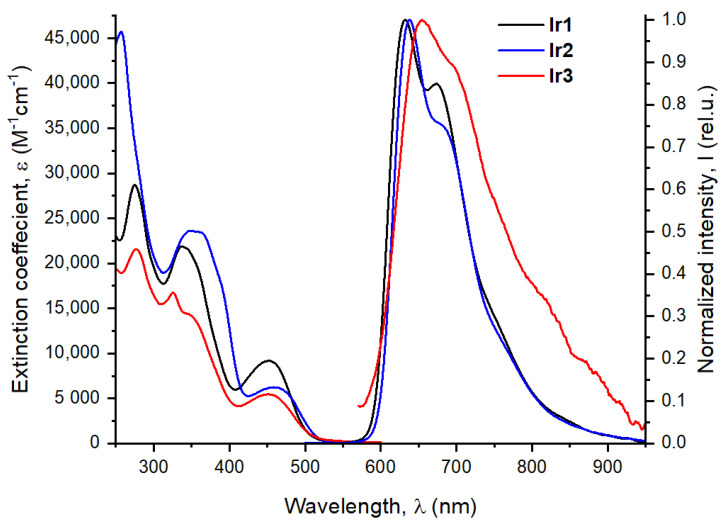
Absorption and emission spectra for complexes **Ir1**–**Ir3** in water (310 K, λ_ex_ 365 nm).

All compounds obtained exhibited luminescence in the red region of the visible spectrum. The absorption and emission spectra of **Ir1**–**Ir3** are shown in Figure 2, and numerical spectroscopic data, together with emission quantum yields and lifetimes in aqueous aerated and deaerated solutions, are summarized in Table 1 and Table S1 (see the Supplementary Materials). The lifetimes were also measured in model physiological media: 0.01M phosphate-buffered saline (PBS) solution (pH = 7.4) containing bovine serum albumin (BSA, 70 µM) and in Dulbecco’s Modified Eagle Medium (DMEM) solution with 10% of fetal bovine serum (FBS).

The absorption spectra of the studied iridium complexes exhibit strong high-energy bands in the range 250–350 nm and low energy absorption at ca. 450 nm with the tail extending below 550 nm. DFT analysis of the absorption spectra (see Tables S3–S8) showed that the observed high-energy bands may be assigned to a combination of the transitions between the aromatic systems of the N^C and N^N ligands with some admixture of the metal to ligand charge transfers (^1^MLCT). The low-energy bands at ca 450 nm may be associated with intraligand and ligand-to-ligand charge transfer (^1^LLCT and ^1^ILCT) localized at two N^C ligands with a minor admixture of ^1^MLCT transitions to N^C to the N^C ligands. The lowest calculated S_0_ ⟶ S_1_ transition, which has a very low oscillator strength, is located well below 500 nm and is associated with electron density transfer to the N^N ligand from N^C ligands and iridium ion.

**Ir1**–**Ir3** demonstrated luminesce in an aqueous solution in the red region of the visible spectrum, showing slightly structured emission bands with the maxima at 632, 638 and 655 nm, respectively; see Figure 2 and Table 1. These complexes displayed large Stokes shifts of ca. 17–200 nm, lifetimes in the microsecond domain and strong sensitivity to the presence of molecular oxygen, is indicative of the triplet nature of the emissive excited state, i.e., phosphorescence. **Ir1** and **Ir2** exhibit rather intense luminescence with the quantum yields 17.3% and 8.5%, respectively, in a deaerated aqueous solution. On the contrary, **Ir3** was a very weak emitter with an emission intensity two orders of magnitude lower compared to its congeners, which makes its application as an oxygen sensor in biological experiments impossible.

**Figure 3:**
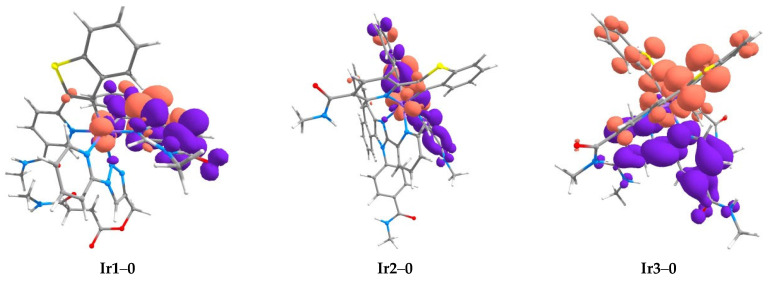
Natural transition orbitals (NTO) for T_1_ ⟶ S_0_ transitions in **Ir1**–**0**, **Ir2**–**0** and **Ir3**–**0**. Violet and terracotta colors show decrease and increase in electron density, respectively. The calculations have been simplified by substitution of OEG pendants for methyl groups in the structure of the complexes. Atom colors: Ir—lilac; S—yellow; O—red; N—blue; C—gray, H—white.

The DFT and TD-DFT calculations gave the emission wavelengths, which were in very good agreement with the experimental data (Table S2). Analysis of the nature of emissive transitions (T_1_ ⟶ S_0_) for the studied complexes showed that the character of **Ir3** emission is essentially different from those revealed for **Ir1** and **Ir2**; see Figure 3 and interfragment charge transfer Tables S4, S6 and S8. In the complexes **Ir1** and **Ir2**, the relaxation processes occur through the ^3^ILCT and ^3^MLCT transitions associated with the N^C cyclometallating ligand. For **Ir3**, the excited-state relaxation is mainly associated with the diimine N^N ligand, and, as a consequence, ^3^MLCT (N^N ⟶ Ir) and ^3^LLCT (N^N ⟶ N^C) transitions are observed. Such a significant difference in the nature of the phosphorescence processes is most probably responsible for the strong difference in emission quantum yield (of the order of 0.1% in deaerated water) of **Ir3** as compared to **Ir1** and **Ir2**. One of the possible explanations of this observation may be the higher contribution of rotational non-radiative channels to excited-state relaxation for **Ir3** due to the presence of two {–C(O)NHR} substituents at the diimine ligand compared to only one substituent at the N^C ligands in **Ir1** and **Ir2**.

**Figure 4:**
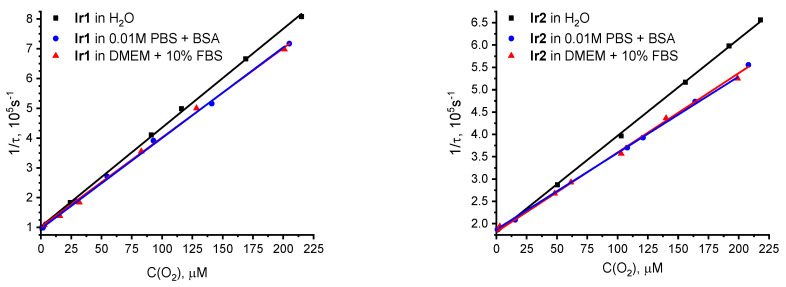
Stern–Volmer oxygen-quenching plots of **Ir1** (**left**) and **Ir2** (**right**) in aqueous solution, 0.01 M PBS (pH 7.4) with 70 µM of BSA and in DMEM with 10% of FBS. T = 37 °C, excitation at 355 nm.

The noticeably higher phosphorescence quantum yields of **Ir1** and **Ir2**, as well as their high sensitivity to the variations in molecular oxygen concentration in solution, led us to choose these compounds for the further studies of their sensor properties and their applicability as luminescent oxygen probes in biosystems. To calibrate the dependence of **Ir1** and **Ir2** excited state lifetimes on oxygen concentration, we carried out the measurements in water and in model biological solutions containing typical components of intracellular media: PBS buffer with the addition of bovine serum albumin—BSA and DMEM with the addition of 10% fetal bovine serum—FBS; see Table 1 and Figure 4. The experiments in the model solutions make it possible to reveal the effect of salinity, pH and the presence of the main protein component of plasma (albumin) on the sensors’ characteristics. The obtained data (Table S1) indicate lifetime independence on pH and media salinity. Aqueous solutions were studied for comparison.

The obtained calibration curves (Figure 4) indicated that the growth (DMEM + 10 % FBS) and model biological media (PBS with 70 mM BSA) had an almost identical effect on the behavior of these sensors. The slope of the Stern–Volmer curves in these media for both complexes coincided under the limits of experimental uncertainty and obviously differed from that in pure water. This observation can be explained by an increase in solution viscosity in the presence of biological macromolecules that reduces the rate of oxygen diffusion in the sample under study and causes a drop in the Stern–Volmer quenching constant. It is also impossible to exclude a reversible non-covalent interaction of the chromophores with hydrophobic pockets of albumin, which may shield the complexes from collisions with oxygen molecules that increase the observed excited state lifetime. The obtained data indicate that the oligo(ethylene glycol) moieties are unable to completely shield the chromophore from the effects of the bio-environment, which in turn implies the use of calibration curves obtained in model physiological media for the estimation of oxygen concentration in biological samples. It is worth noting that **Ir1** and **Ir2** demonstrated high dark stability and low photobleaching rate under irradiation with 355 and 365 nm.

Thus, the obtained data clearly indicate that the phosphorescent complexes **Ir1** and **Ir2** are suitable for application in functional bioimaging as promising oxygen sensors, since they exhibit appreciable quantum yields, high sensitivity to the presence of molecular oxygen and good solubility and stability in aqueous solutions, including model biological media.

### 3.3. Biological Experiments

**Figure 5:**
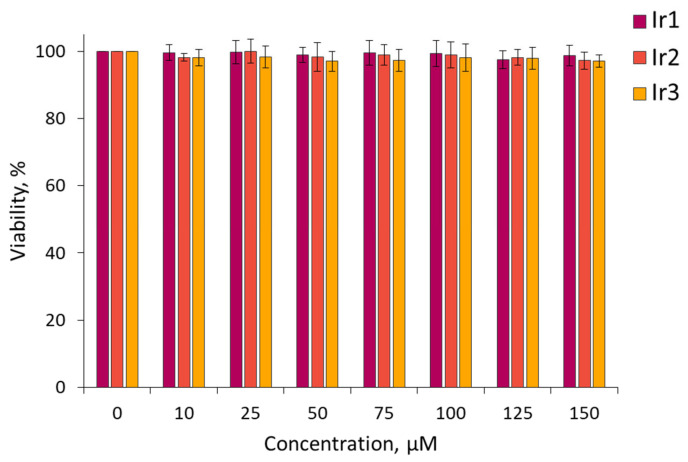
MTT assay for viability of CT26 cells after incubation with **Ir1**–**Ir3** complexes. Mean ± SD. N = 3 repeats by 10 wells.

Using the MTT assay, it was found that all the compounds tested were non-toxic for cultured cancer cells CT26 (murine colorectal carcinoma cell line) and HCT116 (human colorectal carcinoma cell line) in the concentration range studied. Upon incubation with the complexes for 24 h, more than 90% of tumor cells remained viable at concentrations of 150 μM and below (Figure 5 and Figure S24 in Supplementary Materials).

**Figure 6:**
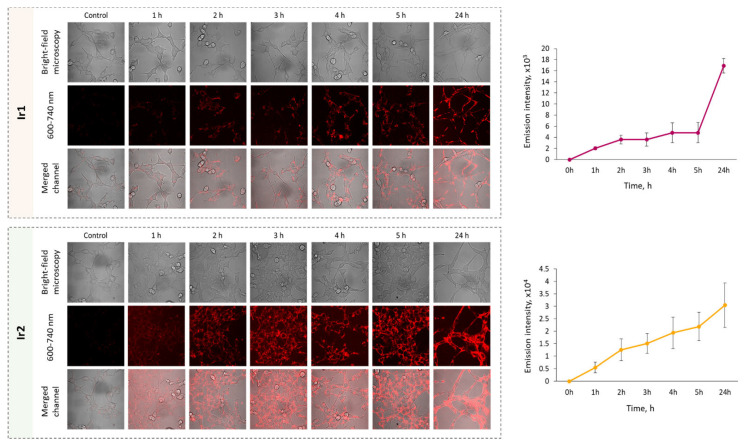
Dynamics of internalization of **Ir1** and **Ir2** complexes into CT26 cells in vitro. Control—cells without the complexes. Mean ± SD. N = 50–70 cells. Scale bar = 50 µm.

Next, the ability of **Ir1**, **Ir2** and **Ir3** to accumulate in living cancer cells was investigated. As mentioned above, **Ir3** featured a very low-emission quantum yield that gave an extremely weak luminescence signal inside cells, thus making this complex unsuitable for further biological testing. Using laser scanning microscopy, it was shown that **Ir1** and **Ir2** successfully penetrated into cultured cancer cells (Figure 6); the phosphorescence intensity of both complexes increased stepwise in the time period from 1 to 24 h of incubation. Complex **Ir2** displayed more intense luminescence compared to **Ir1** under normoxic conditions, due to a higher extinction coefficient at 405 nm (excitation wavelength in cell internalization experiments). Inside the cells, the complexes were distributed heterogeneously as granules in the cytoplasm and also as a homogenous fraction in the cytosol.

**Figure 7:**
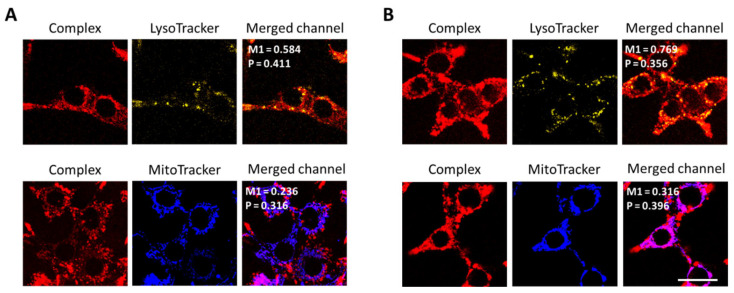
Colocalization of **Ir1** (**A**) and **Ir2** (**B**) complexes with cell organelles. The signal of the complexes was detected in the range 600–740 nm, lysosomes were visualized using a LysoTracker Yellow HCK-123 (detected in the range 520–570 nm) and mitochondria with MitoTracker 405 Blue (detected in the range 420–470 nm). The figures show the Pearson (P) and Manders (M1) colocalization coefficients. Scale bar = 20 µm.

After 3 h of incubation, the subcellular localization of **Ir1** and **Ir2** was analyzed using co-staining with organelle-specific dyes (Figure 7). It was found that both complexes colocalized moderately with lysosomes (M1 = 0.584 for **Ir1** and M1 = 0.769 for **Ir2**) and almost did not colocalize with mitochondria (M1 = 0.236 for **Ir1**, M1 = 0.316 for **Ir2**).

**Figure 8:**
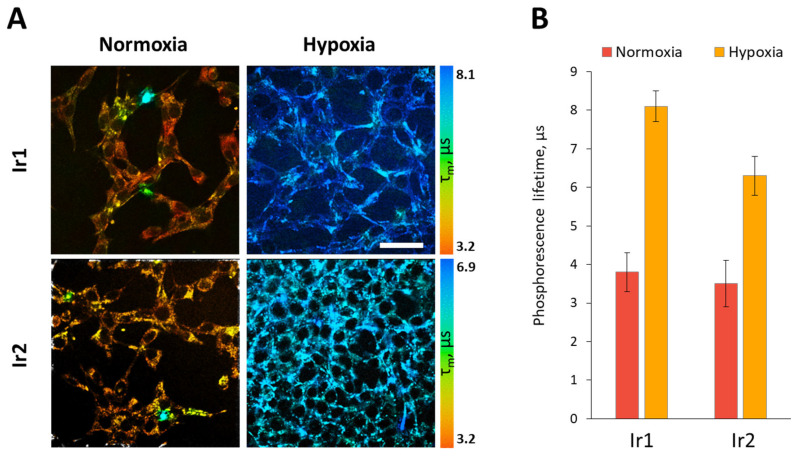
Phosphorescence lifetime variations of **Ir1** and **Ir2** complexes in CT26 cells upon modeling of hypoxic conditions in vitro. (**A**). Representative PLIM images of cells in normoxia and hypoxia. Scale bar = 50 µm. (**B**) Quantification of the phosphorescence lifetime in the cell cytoplasm. Mean ± SD, N = 50 cells.

In order to assess the applicability of the **Ir1** and **Ir2** sensors for determination of the oxygen concentration inside cells, we conducted PLIM experiments, both under conditions of normal oxygenation and hypoxia simulation. In the course of the imaging experiments, we did not observe emission intensity degradation that is indicative of the sensors’ stability in physiological media. These experiments demonstrated that upon modeling hypoxia, the phosphorescence lifetime of the **Ir1** and **Ir2** complexes increased by approximately two times—from 3.8 µs and 3.5 µs to 8.1 µs and 6.3 µs, respectively, indicating the high sensitivity of both complexes localized in cell cytoplasm to variations of oxygen content (Figure 8). It also should be noted that the difference between normoxic and hypoxic phosphorescence lifetimes were slightly more pronounced for **Ir1** (4.3 µs) than for **Ir2** (2.8 µs), and therefore the **Ir1** complex was used for further in vivo testing on mouse tumor models.

After 30 min of local administration of **Ir1** into tumors in vivo at the concentration of 250 µM, the phosphorescence signal was detected primarily in the cytoplasm of tumor cells. Inside the cells, the complex was distributed more homogeneously in comparison with cultured cells in vitro. We also checked the intensity of phosphorescence after 3 h and 6h of local sensor administration (Figure S26 in Supplementary Materials) and did not observe significant changes. Therefore, for greater convenience and reliability of the experiment, we used the smallest incubation interval—30 min.

**Figure 9:**
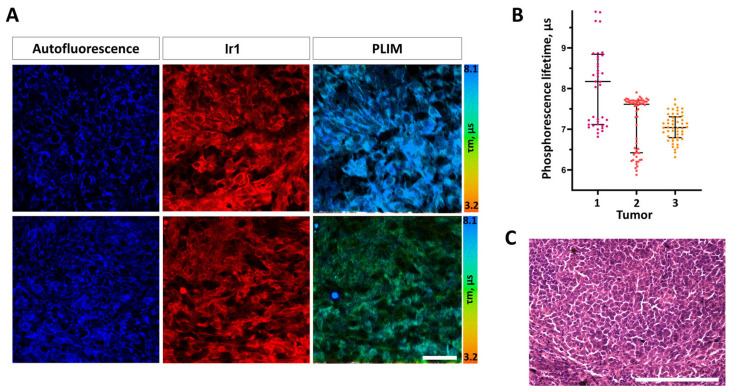
In vivo PLIM of mouse colorectal tumor CT26 with the **Ir1** complex. (**A**) Representative microscopic images of cellular autofluorescence, **Ir1** luminescence intensity and phosphorescence lifetimes in two fields of view. Scale bar = 50 µm. (**B**) Quantification of the phosphorescence lifetime in tumors. Dot plots for three tumors; each dot corresponds to a single cell. Median (Q1; Q3). (**C**) Histological verification of the tumor, H&E. Scale bar = 150 µm.

In CT26 tumor cells in vivo, the phosphorescence lifetime of **Ir1** was 7.4 ± 0.8 μs on average (Figure 9). In HCT116 tumor xenografts, the phosphorescence lifetime of **Ir1** was 8.6 ± 0.5 μs, indicating their more hypoxic status (Figure S25 in Supplementary Materials).

However, within each CT26 tumor, a high heterogeneity of phosphorescence lifetime, and consequently oxygen distribution, was observed at the cellular level. In the same tumors, the phosphorescence lifetimes varied from ~6.8 µs to ~9.7 µs, which, in general, corresponded with the values of the phosphorescence lifetimes typical of different degrees of hypoxia and the lifetime data measured for this complex in cuvette experiments (Table 1).

It is important to note that the dose of the complex used did not induce any acute toxic effect on mice and did not change the typical histological structure of the tumor tissue, thus proving the potential biocompatibility of these phosphorescent O_2_ probes. Therefore, the conducted in vitro and in vivo experiments showed the high potential of the new **Ir1** complex for assessing tissue oxygenation using PLIM.

## 4. Conclusions

We have obtained and comprehensively characterized three new target iridium complexes, [Ir(N^C)_2_(N^N#)]Cl, with various diimine N^N ligands in their composition. These complexes were decorated with short-branched oligo(ethylene glycol) groups to give them water solubility, biocompatibility and low toxicity.

These compounds exhibited oxygen-dependent phosphorescence. The study of their photophysical properties made it possible to determine the two most promising sensors for further biological testing. The quantum yields of these complexes were moderate and reached 17% in deaerated water. The emission wavelengths were in the transparency window of biological tissues.

Biological studies showed that these compounds have low toxicity. Cellular in vitro experiments proved that these sensors exhibit a significant lifetime response to changes in the oxygen concentration in the sample. Simulation of hypoxia in cells led to a two- and threefold increase of these values. The effectiveness of these sensors allowed their application in in vivo experiments on living mice tumor models. In these experiments, the sensors also made it possible to record the presence of significant hypoxia in tumors, as well as their heterogeneity.

Thus, we have obtained and studied very promising new molecular oxygen sensors based on low-toxicity biocompatible phosphorescent iridium complexes.

## Figures and Tables

**Table 1:** Photophysical data for complexes **Ir1**–**Ir3** in aqueous solutions at 310 K.

Medium	Complex	λ_abs_ (nm)	λ_em_ (nm)	Φ_aer_ (%)	Φ_deg_ (%)	τ_aer_ * (µs)	τ_deaer_ * (µs)	τ_deg_/τ_aer_
Water	**Ir1**	275; 355; 352sh; 433sh; 453	632; 674; 760sh	2.1	17.3	1.24	10.2	8.2
PBS + BSA			632; 676; 765sh			1.39	10.0	7.2
DMEM + FBS			633; 677; 765sh			1.43	9.29	6.5
Water	**Ir2**	250sh; 258; 286sh; 346; 368sh; 391sh; 459	638; 689sh; 777sh	2.4	8.5	1.52	5.32	3.5
PBS + BSA			640; 688sh; 780sh			1.80	5.34	3.0
DMEM + FBS			640; 690sh; 780sh			1.90	5.20	2.7
Water	**Ir3**	277; 326; 355sh; 453	655; 700sh; 820sh; 885sh	ca. 0.03	ca. 0.1	0.85	2.88	3.4
PBS + BSA			653; 705sh; 820sh; 880sh			1.74	2.92	1.7
DMEM + FBS			655; 700sh; 820sh; 880sh			1.77	3.04	1.7

Excitation at 365 nm for emission and quantum yield measurements, 355 nm for excitation state lifetime measurements; * independently measured O_2_ concentration is shown in Table S1.

## Data Availability

No data are available.

## Supplementary Materials

The Supplementary Information file and optimized structures of complexes Ir1–0, Ir2–0, Ir3–0 can be downloaded at: https://www.mdpi.com/article/10.3390/bios13070680/s1. References [68,69,70,71,72,73,74,75,76,77,78,79] are cited in the Supplementary Materials. Figure S1: ^1^H NMR spectrum of 6-(benzo[b]thiophen-2-yl)nicotinic acid, (CD_3_)_2_SO, 298 K; Figure S2: ^1^H-^1^H COSY NMR spectrum of 6-(benzo[b]thiophen-2-yl)nicotinic acid, (CD_3_)_2_SO, 298 K; Figure S3: ESI^+^ mass spectrum of 6-(benzo[b]thiophen-2-yl)nicotinic acid ([M+H]^+^cation area), solvent—methanol; Figure S4: 1H NMR spectrum of N^C, CDCl3, 298 K; Figure S5: ^1^H-^1^H COSY NMR spectra of N^C, CDCl_3_, 298 K; Figure S6: ESI^+^ mass spectrum of N^C ([M+Na]^+^cation area), solvent—methanol; Figure S7: ^1^H NMR spectrum of dissociated dimer [Ir_2_(N^C)_4_Cl_2_], (CD_3_)_2_SO, 298 K (top—full view, bottom—aliphatic area); Figure S8: ^1^H-^1^H COSY NMR spectrum of dissociated dimer [Ir_2_(N^C)_4_Cl_2_], (CD_3_)_2_SO, 298 K; Figure S9: ESI^+^ mass spectrum of dissociated dimer ([Ir(N^C)_2_Cl+Na]^+^cation area), solvent—methanol; Figure S10: ^1^H NMR spectrum of **Ir1**, CD_3_OD, 298 K (top—full view, bottom—aliphatic area); Figure S11: ^1^H-^1^H COSY NMR spectrum of **Ir1**, CD_3_OD, 298 K; Figure S12: ESI^+^ mass spectrum of **Ir1** ([M+Na]^2+^cation area), solvent—methanol; Figure S13: ^1^H NMR spectrum of **Ir2**, CD_3_OD, 298 K (top—full view, bottom—aliphatic area); Figure S14: ^1^H-^1^H COSY NMR spectrum of **Ir2**, CD_3_OD, 298 K; Figure S15: ESI^+^ mass spectrum of **Ir2** ([M+Na]^2+^cation area), solvent—methanol; Figure S16: ^1^H NMR spectrum of **Ir3**, CD_3_OD, 298 K (top—full view, bottom—aliphatic area). Peak assignments: * -toluene in CD_3_OD impurities; Figure S17: ^1^H-^1^H COSY NMR spectrum of **Ir3**, CD_3_OD, 298 K; Figure S18: ESI^+^ mass spectrum of **Ir3** ([M+Na]^2+^cation area), solvent—methanol; Figure S19: Absorption spectra of **Ir1**: experimental (red) and calculated (black) lines with oscillator strengths of electronic transitions (bars); Figure S20: Absorption spectra of **Ir2**: experimental (red) and calculated (black) lines with oscillator strengths of electronic transitions (bars); Figure S21: Absorption spectra of **Ir3**: experimental (red) and calculated (black) lines with oscillator strengths of electronic transitions (bars); Figure S22: Optimized structure of the model **Ir2**–**0** complex (hydrogen atoms are omitted for clarity). The calculations have been simplified by substitution of OEG pendants in **Ir2** complex for methyl groups in **Ir2**–**0** structure. Atom colors: Ir—blue; S—yellow; O—green; N—red; C—gray; Figure S23: Optimized structure of the model **Ir3**–**0** complex (hydrogen atoms are omitted for clarity). The calculations have been simplified by substitution of OEG pendants in **Ir3** complex for methyl groups in **Ir3**–**0** structure. Atom colors: Ir—blue; S—yellow; O—green; N—red; C—gray; Figure S24: MTT assay for viability of HCT116 cells after incubation with complexes **Ir1**–**Ir3**. Mean ± SD. N = 3 repeats by 10 wells; Figure S25: In vivo PLIM of human colorectal tumor HCT116 with the **Ir1** complex. Representative microscopic images of cellular autofluorescence, **Ir1** luminescence intensity, PLIM (scale bar = 50 µm.) and histological verification (scale bar = 150 µm); Figure S26: In vivo PLIM of mouse colorectal tumor CT26 with the **Ir1** complex. Representative microscopic images of **Ir1** luminescence intensity, PLIM at 30 min, 3 h and 6 h after sensor injection. Scale bar = 50 µm; Table S1: Values of excitation state lifetime of complexes **Ir1** and **Ir2** in various aqueous media and at different oxygen concentrations. Excitation at 355 nm, T = 37 °C, C (Complex) = 10 µM; Table S2: Calculated and experimental wavelength of emission maxima of complexes **Ir1**–**Ir3**; Table S3: Calculated absorption maxima (λ) and oscillator strengths (f) of complex **Ir1**; Table S4: Natural transition orbitals (NTO) for **Ir1**–**0**; violet and terracotta colors show decrease and increase in electron density, respectively. The calculations have been simplified by substitution of OEG pendants of **Ir1** complex for methyl groups in **Ir1**–**0** structure. Atom colors: Ir—lilac; S—yellow; O—red; N—blue; C—gray; H—white. The data for the corresponding interfragment charge transfer (IFCT) are given below the figures. Diagonal values represent intraligand transitions; off-diagonal values represent a charge transfer from “Donor” to “Acceptor”; Table S5: Calculated absorption maxima (λ) and oscillator strengths (f) of complex **Ir2**; Table S6: Natural transition orbitals (NTO) for **Ir2**–**0**; violet and terracotta colors show decrease and increase in electron density, respectively. The calculations have been simplified by substitution of OEG pendants of **Ir2** complex for methyl groups in **Ir2**–**0** structure. Atom colors: Ir—lilac; S—yellow; O—red; N—blue; C—gray; H—white. The data for the corresponding interfragment charge transfer (IFCT) are given below the figures. Diagonal values represent intraligand transitions; off-diagonal values represent a charge transfer from “Donor” to “Acceptor”; Table S7: Calculated absorption maxima (λ) and oscillator strengths (f) of complex **Ir3**; Table S8: Natural transition orbitals (NTO) for **Ir3**–**0**; violet and terracotta colors show decrease and increase in electron density, respectively. The calculations have been simplified by substitution of OEG pendants of **Ir3** complex for methyl groups in **Ir3**–**0** structure. Atom colors: Ir—lilac; S—yellow; O—red; N—blue; C—gray; H—white. The data for the corresponding interfragment charge transfer (IFCT) are given below the figures. Diagonal values represent intraligand transitions; off-diagonal values represent a charge transfer from “Donor” to “Acceptor”; Table S9: The key structural parameters of complex **Ir1**–**0** (R = NHMe) and its literature analog; Table S10: The key structural parameters of complex **Ir2**–**0** (R = NHMe) and its literature analog; Table S11: The key structural parameters of complex **Ir3**–**0** (R = NHMe) and its literature analog.
