# Supplementary material for: Biocompatible Phosphorescent O2 Sensors Based on Ir(III) Complexes for In Vivo Hypoxia Imaging
Source: Biosensors (Basel). 2023 Jun 26;13(7):680. doi: 10.3390/bios13070680 (PMC10377268; doi:10.3390/bios13070680)
Supplement: Supplementary file 1 [file biosensors-13-00680-s001.zip › biosensors-2413506-supplementary.pdf]

biosensors

MDPI

Article

Biocompatible Phosphorescent O_2 Sensors Based on Ir(III) Complexes for In Vivo Hypoxia Imaging

Mozhgan Samandarsangari [1], Daria O. Kozina [1], Victor V. Sokolov [1], Anastasia D. Komarova [2,3], Marina V. Shirmanova [2], Ilya S. Kritchenkov [1,*] and Sergey P. Tunik [1,*]

1. Synthesis of Ligands and Complexes

Synthesis of 6-(benzo[b]thiophen-2-yl)nicotinic acid. Methyl 6-(benzo[b]thiophen-2-yl)nicotinate (400.0 mg, 1.485 mmol) and KOH (250.0 mg, 4.455 mmol) were stirred in THF/H_2O (1:1, 6 mL) at RT for 90 h. The mixture was diluted with water (60 mL) and washed with $CHCl_3$ (60 mL). After two $CHCl_3$ washings, the aqueous layer was separated, neutralized with 1 M $HCl_{(aq)}$, concentrated in vacuum (to 30 mL) and acidified to pH = 5. The precipitate was collected by filtration, washed with water and dried in vacuum to afford the title compound as a pale yellow solid, requiring no further purification. Yield: 285 mg (75%). ^{1}H NMR (($(CD_3)_2SO$, 400 MHz, δ): 9.08 (s, 1H), 8.34 (d, J = 8.3 Hz, 1H), 8.32 (s, 1H), 8.23 (d, J = 8.3 Hz, 1H), 8.03-8.00 (m, 1H), 7.94-7.92 (m, 1H), 7.45-7.41 (m, 2H). HRMS (ESI) m/z: 256.0432 calculated for $C_{14}H_{10}NO_2S^+$ [M+H]$^+$, found 256.0386.

Synthesis of N^C ligand. A suspension of 6-(benzo[b]thiophen-2-yl)nicotinic acid (125.0 mg, 0.490 mmol), 2,5,8,12,15,18-hexaoxanonadecan-10-amine (173.7 mg, 0.588 mmol), N,N′ dicyclohexylcarbodiimide (DCC, 121.3 mg, 0.588 mmol), N-hydroxysuccinimide (NHS, 67.7 mg, 0.588 mmol), 4-(dimethylamino) pyridine (DMAP, 1.8 mg, 0.015 mmol), NEt_3 (59.5 mg, 0.588 mmol) in dry DMSO (1.0 mL) was stirred at 40 °C for 4 days. The reaction mixture was concentrated in vacuum, the residue was suspended in 5 mL acetonitrile, cooled for 2 days and the solution was isolated by centrifugation and vacuum-dried to give the oily substance. 2 mL of water was added to the oily residue, sonicated, the aqueous solution was decanted and the residue was vacuum dried. The oily residue was dissolved in DCM, cooled for 1 day, centrifuged and the solution was thoroughly dried to give the desired compound. Yield: 231 mg (89%).^{1}H NMR ($CDCl_3$, 400 MHz, δ): 9.02 (s, 1H), 8.19 (d, J = 8.3 Hz, 1H), 7.91 (s, 1H), 7.88-7.81 (m, 3H), 7.39-7.34 (m, 2H), 7.04 (d, J = 8.1 Hz, NH), 4.45 (m, 1H, CH), 3.81-3.51 (m, 20H, CH_2), 3.35 (s, 6H, CH_3). HRMS (ESI) m/z: 555.2141 calculated for $C_{27}H_{36}N_2NaO_7S^+$ [M+Na]$^+$, found 555.2105.

Synthesis of iridium dimeric complex [$Ir_2(N\hat{}C_2)_4Cl_2$]. $IrCl_3{\cdot}6H_2O$ (88.2 mg, 0.217 mmol), N^C ligand (231 mg, 0.434 mmol), a mixture of distilled 2-methoxyethanol (10 mL) and water (2.5 mL) were placed in a 25 mL test tube with screw cap and degassed for 20 minutes. The reaction mixture was stirred at 100 °C for 18 h. The resulting dark-red solution was thoroughly evaporated, the dried substance was dissolved in 0.4 mL of $CHCl_3$ and precipitated by adding 3 mL of n-hexane. The resulting suspension was sonicated, decanted, and the oily residue was vacuum dried. The residue was washed with water twice (2 × 1 mL) and vacuum dried. The product was dissolved in 1 mL of DCM, cooled for 1 h, centrifuged and the solution was evaporated. The resulting solid was dissolved in 0.3 mL of DCM and precipitated by adding 2 mL of Et_2O and then 1 mL of n-hexane, solicited, decanted, the precipitate was dried under reduced pressure to give dark-red solid. Yield: 260 mg (93%).^{1}H NMR ($(CD_3)_2SO$, 400 MHz, δ): 10.39 (s, 1H), 10.11 (s, 1H), 8.74 (d, J = 8.0 Hz, 1H, NH), 8.65 (d, J = 8.0 Hz, 1H, NH), 8.60 (d, J = 8.4 Hz, 1H), 8.51 (d, J = 8.4 Hz, 1H), 8.04 (d, J = 8.4 Hz, 1H), 7.89 (d, J = 8.4 Hz, 1H), 7.86 (d, J = 8.1 Hz, 1H), 7.81 (d, J = 8.2 Hz, 1H), 7.22 (t, H = 7.7 Hz, 1H), 7.15 (t, J = 7.7 Hz, 1H), 6.92 (t, J = 7.7 Hz, 1H), 6.78 (t, J = 7.7 Hz, 1H), 6.25 (d, J = 8.1 Hz, 1H), 5.56 (d, J = 8.3 Hz, 1H), 4.30 (m, 2H, CH), 3.63-3.42 (m, 40H, CH_2), 3.23 (s, 9H, CH_3), 3.22 (s, 3H, CH_3). HRMS (ESI) m/z: 1313.3537 calculated for $C_{54}H_{70}ClIrN_4NaO_{14}S_2^+$ [Ir(N^C)$_2$Cl+Na]$^+$, found 1313.3737.

General procedure for the synthesis of iridium complexes Ir1, Ir2 and Ir3. To obtain [Ir(N^C)$_2$(N^N#)]Cl complexes, the [$Ir_2(N\hat{}C_2)_4Cl_2$] dimeric complex (50 mg, 0.019 mmol), was added to the 2 mL methanol solution of the corresponding N^N# ligand (0.040 mmol) and reacted for 20 h at RT. Then the solutions were dried under reduced pressure and worked up as follows.

Complexes Ir1 and Ir2. The resulting residues were dissolved in 0.3 mL of acetone and then precipitated by adding of 3.0 mL of Et_2O, solicited, decanted and dried. The residues were dissolved in 1.5 mL of water, centrifuged, and the aqueous solutions were vacuum dried. The dried residues were dissolved in 1 mL of ethyl acetate, centrifuged and the solvents were evaporated in vacuo, and the products were purified by silica gel

column chromatography ($CHCl_3$/methanol 20:1, then 10:1 and then 5:1 mixture). The target fractions were combines, dried in vacuum, and the obtained solid residues were dissolved in 0.3 mL of acetone and then precipitated by adding of 3.0 mL of Et_2O and 1 mL of n-hexane, sonicated, decanted and the precipitates were thoroughly dried to obtain the final Ir(III) complexes.

Complex Ir1. Yield: 50 mg (72%). 9.53 (s, 1H), 8.57 (d, J = 8.3 Hz, 1H), 8.44-8.38 (m, 3H), 8.05 (s, 1H), 7.99 (s, 1H), 7.95-7.89 (m, 3H), 7.83 (d, J = 8.3 Hz, 1H), 7.77 (d, J = 8.3 Hz, 1H), 7.64 (t, J = 7.5 Hz, 1H), 7.24 (t, J = 7.6 Hz, 1H), 7.20 (t, J = 7.6 Hz, 1H), 6.90 (t, J = 7.7 Hz, 1H), 6.86 (t, J = 7.7 Hz, 1H), 6.10 (d, J = 8.2 Hz, 1H), 6.03 (d, J = 8.2 Hz, 1H), 5.30 (s, 2H, CH_2), 4.34-4.23 (m, 2H, CH), 4.14-4.08 (m, 1H, CH), 3.64-3.44 (m, 60H, CH_2), 3.32 (s, 3H, CH_3), 3.31 (s, 3H, CH_3), 3.30 (s, 6H, CH_3), 3.29 (s, 6H, CH_3). HRMS (ESI) m/z: 915.8300 calculated for $C_{79}H_{109}IrN_9NaO_{23}S_2^{2+}$ $[M+Na]^{2+}$, found 915.8296.

Complex Ir2. Yield: 72 mg (95%). 9.37 (s, 1H), 8.70 (d, J = 8.0 Hz, 1H), 8.51 (d, J = 8.5 Hz, 2H), 8.46-8.39 (m, 3H), 8.23 (d, J = 8.2 Hz, 1H), 8.18 (s, 1H), 8.15 (d, J = 8.5 Hz, 1H), 8.09 (d, J = 8.6 Hz, 1H), 7.96 (d, J = 8.2 Hz, 1H), 7.91-7.89 (m, 1H), 7.87-7.83 (m, 1H), 7.78 (t, J = 8.1 Hz, 2H), 7.60 (t, J = 7.6 Hz, 1H), 7.44 (d, J = 8.3 Hz, 1H), 7.34-7.23 (m, 4H), 7.18 (t, J = 7.6 Hz, 1H), 7.12-7.09 (m, 3H), 6.81 (t, J = 7.6 Hz, 1H), 6.52 (d, J = 8.2 Hz, 1H), 6.13 (d, J = 8.3 Hz, 1H), 5.81 (t, J = 7.6 Hz, 1H), 4.55 (m, 1H, CH), 4.40 (m, 1H, CH), 4.20 (m, 1H, CH), 3.83-3.38 (m, 60H, CH_2), 3.33 (s, 6H, CH_3), 3.26 (s, 3H, CH_3), 3.25 (s, 3H, CH_3), 3.23 (s, 3H, CH_3), 3.06 (s, 3H, CH_3). HRMS (ESI) m/z: 985.3532 calculated for $C_{94}H_{114}IrN_8NaO_{21}S_2^{2+}$ $[M+Na]^{2+}$, found 985.3558.

Complex Ir3. The obtained solid was dissolved in 0.3 mL of DCM and then precipitated by adding of 3.0 mL of Et_2O and 1 mL of n-hexane, sonicated, decanted and the precipitate was thoroughly dried. The residue was dissolved in 1.2 mL of water, centrifuged, and the aqueous solution was vacuum dried. The dried residue was dissolved in 1 mL of ethyl acetate, centrifuged and dried. This solid was dissolved in 1 mL of DCE, centrifuged and the solution was dried under vacuum. The resulting red solid was dissolved in 0.2 mL of DCM and then precipitated by adding of 3.0 mL of Et_2O, sonicated, decanted and vacuum dried and this process was repeated six times. Then the solid residue was dissolved in 1 mL of acetonitrile, centrifuged and the solution was dried. Further, the solid was dissolved in 1 mL of toluene, centrifuged and the solution was thoroughly dried. Dried residue was dissolved in 0.2 mL of acetone and precipitated by adding 4 mL of Et_2O, sonicated, decanted and vacuum dried. Yield: 47 mg (59%). 9.24 (s, 1H), 8.38 (d, J = 8.5 Hz, 1H), 8.15 (d, J = 5.6 Hz, 1H), 8.06 (s, 1H), 7.99 (d, J = 5.6 Hz, 1H), 7.93 (d, J = 8.5 Hz, 1H), 7.86 (d, J = 8.1 Hz, 1H), 7.25 (t, J = 7.6 Hz, 1H), 6.90 (t, J = 7.6 Hz, 1H), 6,13 (d, J = 8.1 Hz, 1H), 4.42 (m, 1H, CH), 4.23 (m, 1H, CH), 3.71-3.45 (m, 40H, CH_2), 3.30 (s, 3H, CH_3), 3.28 (s, 3H, CH_3), 3.21 (s, 3H, CH_3), 3.20 (s, 3H, CH_3). HRMS (ESI) m/z: 1038.4058 calculated for $C_{92}H_{132}IrN_8NaO_{28}S_2^{2+}$ $[M+Na]^{2+}$, found 1038. 4411.

2. Experimental details

Photophysical experiments. Photophysical measurements were performed in aqueous media. Absorption spectra were recorded using Shimadzu UV-1800 spectrophotometer. The emission spectra were measured on Avantes AvaSpec-2048x64 spectrometer. The values of the absolute emission quantum yield in solution were measured using a comparative method, 365 nm LED as excitation source and $[Ru(bpy)_3][PF_6]_2$ water solution (Φ = 0.040 air-saturated, 0.063 Ar-saturated) as the reference. For the lifetime measurements pulse laser DTL-375QT (wavelength 355 nm, pulse width 5 ns, repetition frequency 1000 Hz), Hamamatsu (H10682-01) photon counting head, FASTComTec (MCS6A1T4) multiple-event time digitizer and Ocean Optics monochromator (Monoscan-2000, interval of wavelengths 1 nm) were used. PyroScience FireStingO2 oxygen meter, equipped with an oxygen sensor OXROB10 and a temperature sensor TDIP15, was used to measure concentration and partial pressure of molecular oxygen in aqueous solutions. Quantum Northwest qpod-2e cuvette sample compartment was used for temperature control.

Computational Details. Optimized geometry of ground and exited triplet states for all complexes under consideration and their photophysical properties were calculated using the Gaussian 16[1] computer code with DFT methodology. For these purposes, the hybrid Austin-Frisch-Petersson functional with dispersion (APFD)[2] was used due to their best agreement with the experimental results. For all atoms were chosen the Stuttgart-Dresden effective core pseudopotential (SDD) with the corresponding basis set[3]. The Polarizable Continuum Model (PCM)[4] was applied for simulation of effects of water as a non-specific solvent.

Energies of emission were calculated as the difference between the energy of the optimized triplet state and the energy of singlet state at the same triplet geometry ('vertical' transition). The electronic absorption spectra were obtained using TD-DFT methodology with 180 excited states for all complexes. The UV/Vis absorption spectra was obtained from previously calculated oscillator strengths using the method described in work[5] with Lorentzian broadening of 2000 cm^{-1}.

The electron density during absorption and emission transitions was investigated using Natural Transition Orbitals (NTO)[6] and Interfragment Charge Transfer (IFCT)[7] calculations. The Multiwfn 3.6 software[7] was used for both methods. The changes of electronic density $\Delta\varrho$ during the $S_0 \rightarrow S_i$ transitions were estimated as:

$$\Delta\rho(S_0 \rightarrow S_i) = \sum_k |\Psi_{ik}(virt)|^2 - \sum_k |\Psi_{ik}(occ)|^2$$

where $\Psi_{ik}(occ)$ and $\Psi_{ik}(virt)$ are NTO pairs for $S_0 \rightarrow S_i$ transition. The electronic density's change during $T_1 \rightarrow S_0$ transition was similarly.

Cell cultures. The experiments were carried out on cell lines CT26 (mouse colorectal cancer) and HCT116 (human colorectal cancer) that had passed at least 3 and no more than 6 passages. The cells were cultured according to the standard procedure in the complete growth medium DMEM (Gibco, Carlsbad, CA, USA) supplemented with glucose (4.5 g/l) (Gibco, Carlsbad, CA, USA), L-glutamine (Gibco, Carlsbad, CA, USA) and 10% fetal bovine serum (HyClone, USA) at 37°C, 5% CO_2. Cell passage was performed 3 times a week using Versen solution (Gibco, Carlsbad, CA, USA) and 0.25% trypsin-EDTA (Thermo Fisher Scientific, Waltham, MA, USA), when the cell monolayer filled 70–90% of the area of the culture flask.

For the MTT assay, cells were seeded in 96 well plates: 2000 CT26 cells or 10,000 HCT116 cells per well in 200 µl of DMEM. For confocal microscopy, CT26 cells were seeded in 35 mm glass bottom FluoroDish dishes (Ibidi GmbH, Gräfelfing, Germany) at 300,000 cells in 2 ml of DMEM.

For biological experiments, solutions of the studied sensors were prepared. To prepare a stock solution with a concentration of 1 mM, we used water for injection and weighed samples of sensors calculated based on molecular weight.

Solutions for MTT assay and for experiments in vitro (75 μM) was prepared from a stock solution (1 mM) by dissolving in a DMEM with the addition of 10% fetal bovine serum.

Solution for experiments in vivo (250 μM) was prepared from stock solution (1 mM) by dissolving in water for injection.

Tumor Model. All animal protocols were approved by the Local Ethical Committee of the Privolzhsky Research Medical University (Nizhny Novgorod, Russia). Experiments were performed on female Balb/C mice (n = 3) and nu/nu mice (n = 2) weighing 20–22 g. Tumor cells were inoculated in PBS solution intradermally into the mouse ear in the amount of 200,000 CT26 cells or 500,000 HCT116 cells. The study was carried out on the 10-12th day after the cells inoculation, when the tumors were 6-8 mm in diameter. Mice were anesthetized by an intramuscular injection of Zoletil (40 mg/kg, Virbac, Carros, France) and 2% Rometar (10 mg/kg, Spofa, Jicin, Czech Republic). After PLIM experiment, the tumors were excised and placed in a 10% formalin for pathomorphological analysis.

MTT-assay. Ir1, **Ir2** and **Ir3** were added to the cells at the concentrations of 10 μM, 25 μM, 50 μM, 75 μM, 100 μM, 125 μM, and 150 μM and incubated for 24 hours at 37°C, 5% CO_2. After that, the medium was replaced with the MTT reagent solution at a concentration of 0.5 mg/ml, according to the manufacturer's protocol (Alfa Aesar, Haverhill, MA, USA). After 4-hours incubation, the MTT reagent was replaced with a DMSO solution (100 μl per well) to dissolve the formazan crystals. Optical density was measured using a multimodal plate reader (Synergy Mx; BioTek Instruments, Winooski, VT, USA). For each well, the percentage of viable cells relative to the control was determined. For each concentration of the complex, 3 replications of 10 wells were performed.

Laser scanning microscopy. The studies were carried out on the LSM 880 (Carl Zeiss, Berlin, Germany) equipped with a PLIM module based on time-correlated single photon counting TCSPC (Becker&Hickl GmbH, Germany), and a Mai-Tai femtosecond Ti:Sa laser (Spectra Physics, Milpitas, CA, USA) with a pulse repetition rate of 80 MHz and a duration of 120 fs. All images were obtained using a water-immersion lens C-Apochromat 40×/1.2 NA.

In cellular accumulation and distribution assays, the phosphorescence of the **Ir1** and **Ir2** complexes was excited in one-photon mode at a wavelength of 405 nm using a pulse diode laser, and the emission was detected in the range of 600–740 nm. The intensity images were obtained after incubation of CT26 cells with the 75 μM solutions of the complexes for 1, 2, 3, 4, 5 and 24 hours. Before imaging, cells were washed with PBS and placed in FluoroBright DMEM (Thermo Fisher Scientific, USA) containing 10% FBS (Gibco, Carlsbad, CA, USA). Cells without the complexes were used as a control. The emission intensity of the complexes was measured in the cytoplasm of individual cells using ROI option in ImageJ software (National Institutes of Health, Bethesda, MY, USA).

Cell organelles were stained with LysoTracker Yellow HCK-123 (Thermo Fisher Scientific, Waltham, MA, USA) and MitoTracker 405 Blue (Thermo Fisher Scientific, Waltham, MA, USA). Cells were incubated with 75 μM solutions of the **Ir1** and **Ir2** for 3 hours, washed with PBS and then stained with the organelle-specific dyes (LysoTracker: 3 μM, 1 hour incubation; MitoTracker: 50 nM, 15 minutes incubation), according to the manufacturer's protocols.

The fluorescence of LysoTracker was excited at a wavelength of 488 nm and detected in the range of 520–570 nm, that of MitoTracker - at 405 nm and at 420–470 nm, phosphorescence of complexes – at 405 nm and detected in the range 600-740 nm, correspondingly. Images of cells in transmitted light channel were recorded to observe cell morphology. The colocalization of the **Ir1** and **Ir2** complexes and organelle-specific dyes was assessed using the Jacob plugin in ImageJ to determine the Pearson's (P) and Manders'(M1) colocalization coefficients.

PLIM *in vitro* and *in vivo*. Phosphorescence was excited at a wavelength of 750 nm (two-photon excitation) and detected in the range 495–690 nm. Laser power on a sample

was 6 mW. The accumulation time of the phosphorescence signal was 120 s. The field of view was 256x256 μm in size.

For PLIM measurements, the cells were incubated with 75 μM of **Ir1** and **Ir2** for 3 hours in a CO_2 incubator (37°C, 5% CO_2). PLIM images were recorded in normoxic conditions in a stage top incubator (37°C, 5% CO_2) or upon simulated hypoxia. To model hypoxia *in vitro*, a cover slip was placed on the monolayer of cancer cells for 1.5 hours[8]. PLIM images of 4-5 microscopic fields of view were recorded in each culture dish.

The protocol of PLIM *in vivo* was adopted from literature[9]. Briefly, the **Ir1** sensor was injected locally into the tumor at a concentration of 250 μM (in 100 μl) 30 minutes before PLIM. A skin flap over the tumor was surgically opened in sterile conditions, the mouse was placed on a glass coverslip and the ear with a tumor was secured with surgical tape. The search of the regions of interest was performed by detecting cellular autofluorescence upon two-photon excitation at 750 nm, and signal registration at 450-490 nm. After focusing on the cell-rich area, the microscope was switched in PLIM mode. In a total, 5-7 PLIM images were obtained from each tumor.

Processing of the PLIM images was performed using SPCImage 8.5 software (Becker & Hickl GmbH, Germany). Monoexponential phosphorescence decay curve were approximated by the least squares method. Phosphorescence signal was analyzed in the cytoplasm of the individual cells by selecting the cell area without nucleus as a region of interest. For each tumor, 30-50 cells were analyzed. The average number of photons was 1500-2000 in a pixel, so that the binning factor 4 was applied to build a decay curve.

Histopathological analysis. To verify tumors, the tissue samples were embedded in paraffin and histological 7 μm thickness sections were obtained and stained with hematoxylin-eosin. Microscopic images of histological sections were taken using a Leica DFC290 microscope (Leica Microsystems, Wetzlar, Germany) at x20 magnification.

3. NMR and ESI HRMS data

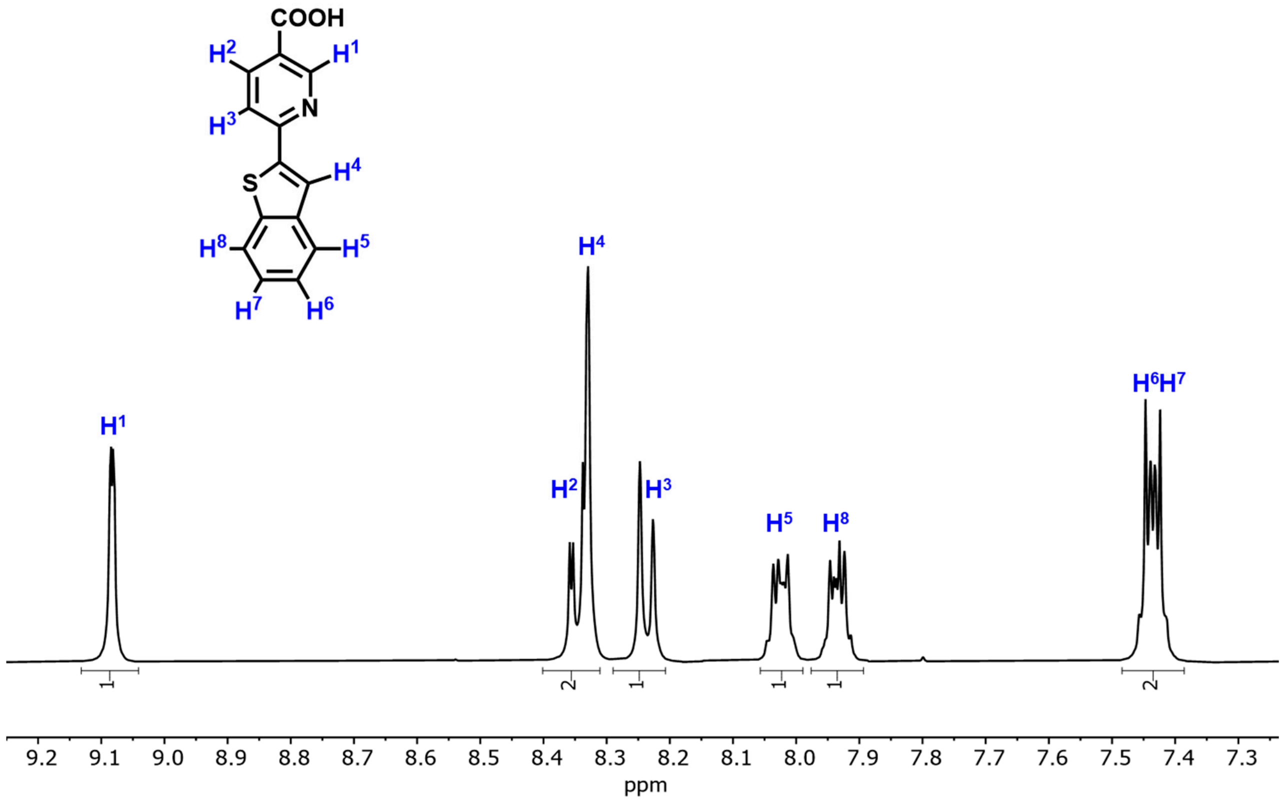

Figure S1. ^{1}H NMR spectrum of 6-(benzo[b]thiophen-2-yl)nicotinic acid, $(CD_3)_2SO$, 298 K.

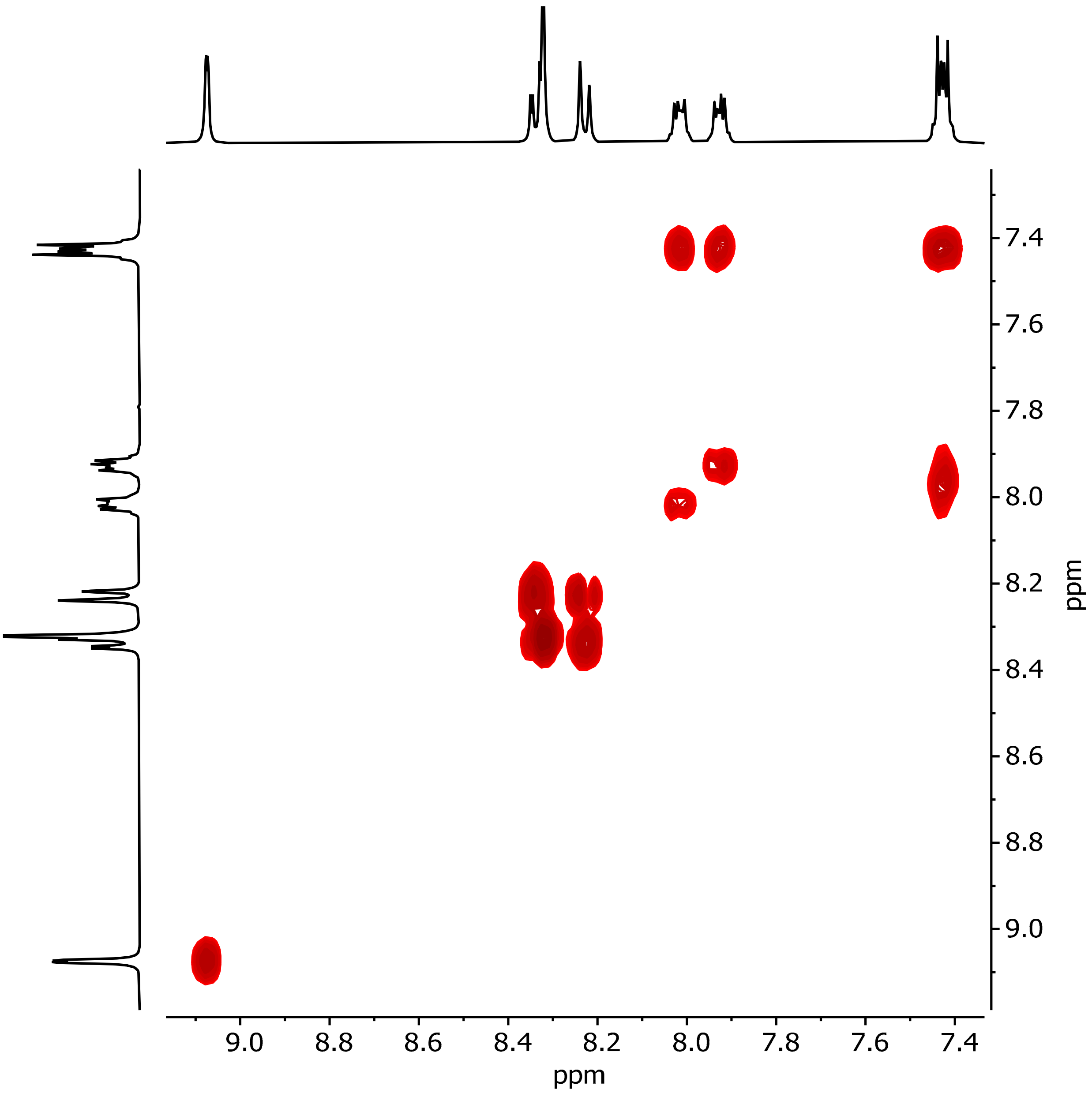

Figure S2. ^{1}H-^{1}H COSY NMR spectrum of 6-(benzo[b]thiophen-2-yl)nicotinic acid, $(CD_3)_2SO$, 298 K.

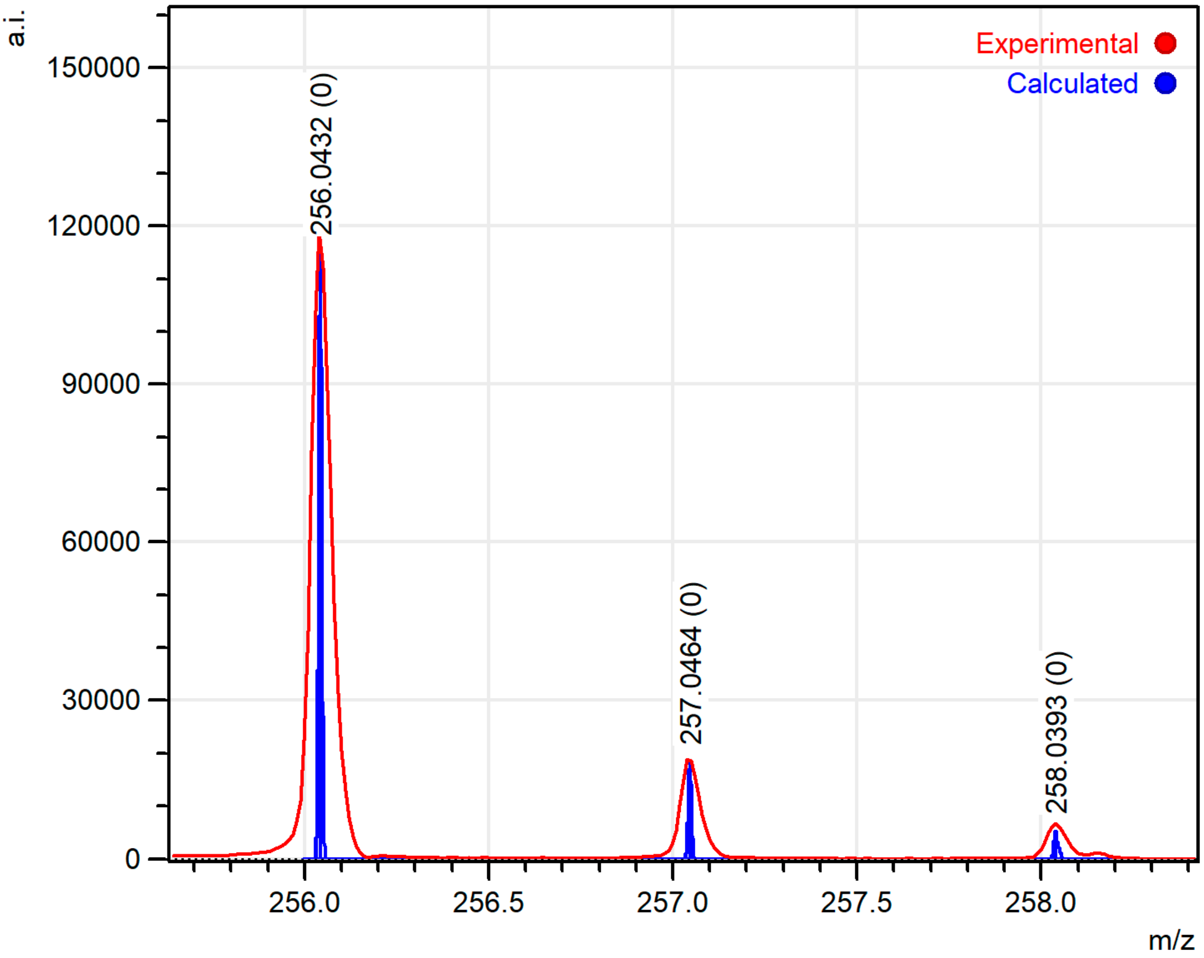

Figure S3. ESI⁺ mass-spectrum of 6-(benzo[b]thiophen-2-yl)nicotinic acid ([M+H]⁺cation area), solvent – methanol.

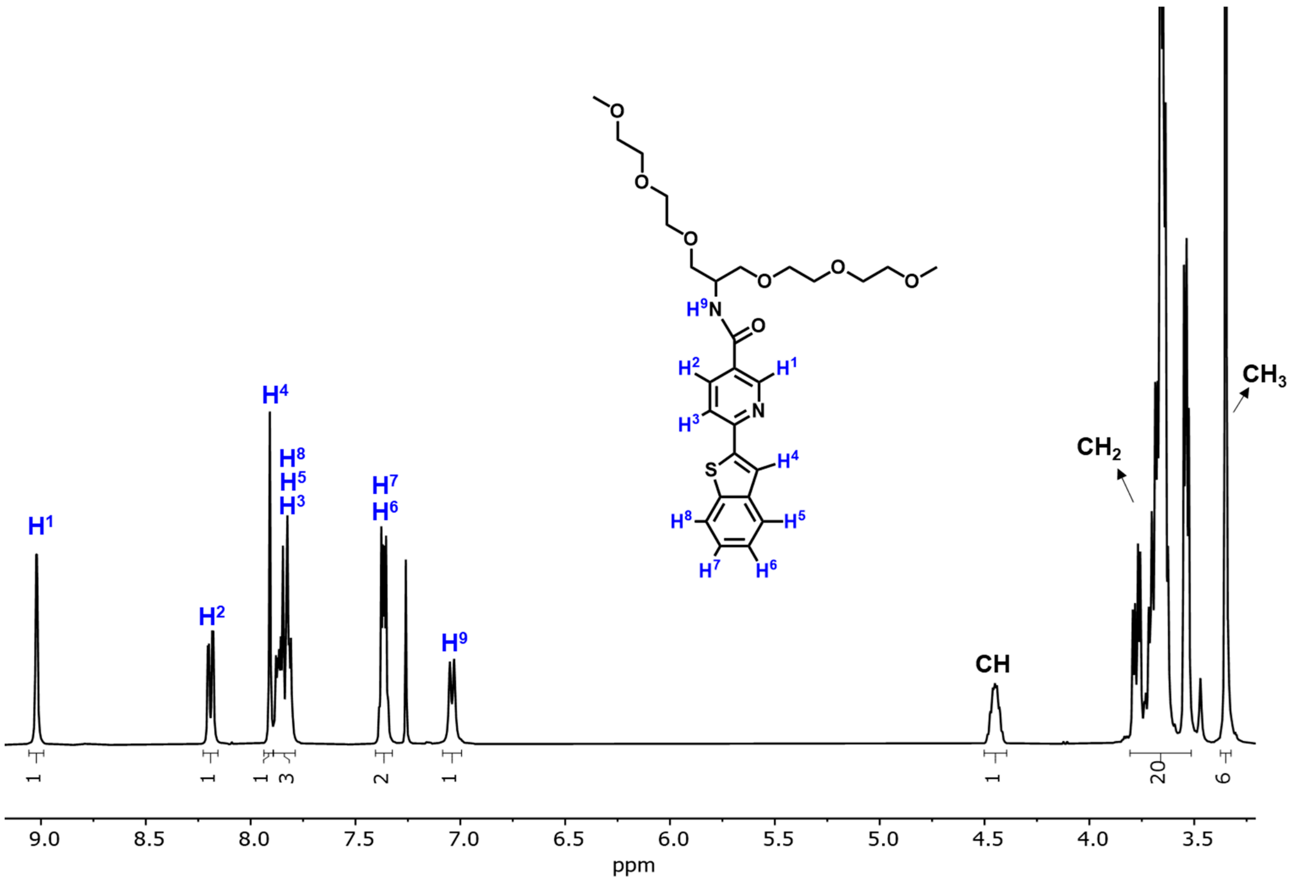

Figure S4. ^{1}H NMR spectrum of N^C, $CDCl_3$, 298 K.

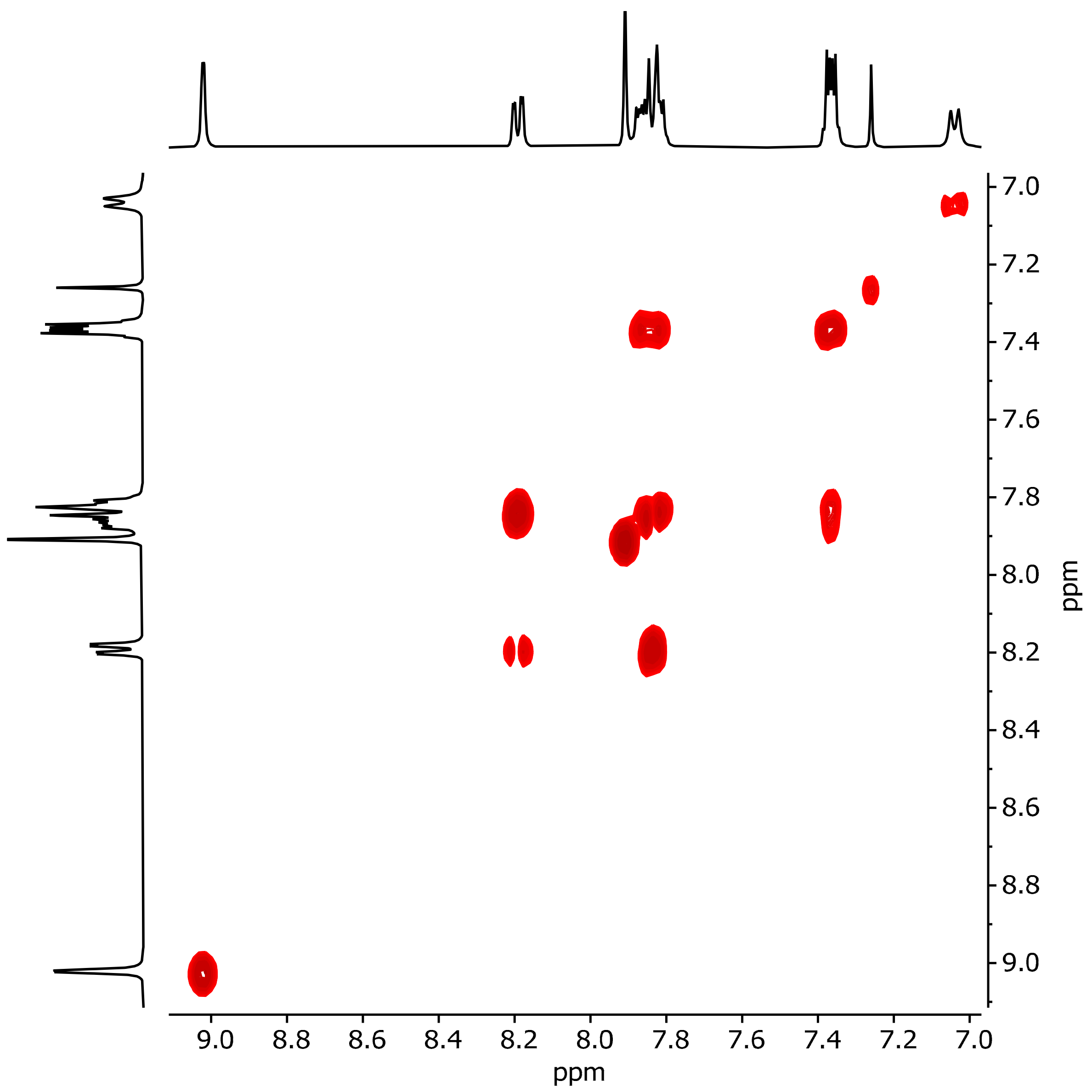

Figure S5. ^1H-^1H COSY NMR spectra of N^C, $CDCl_3$, 298 K.

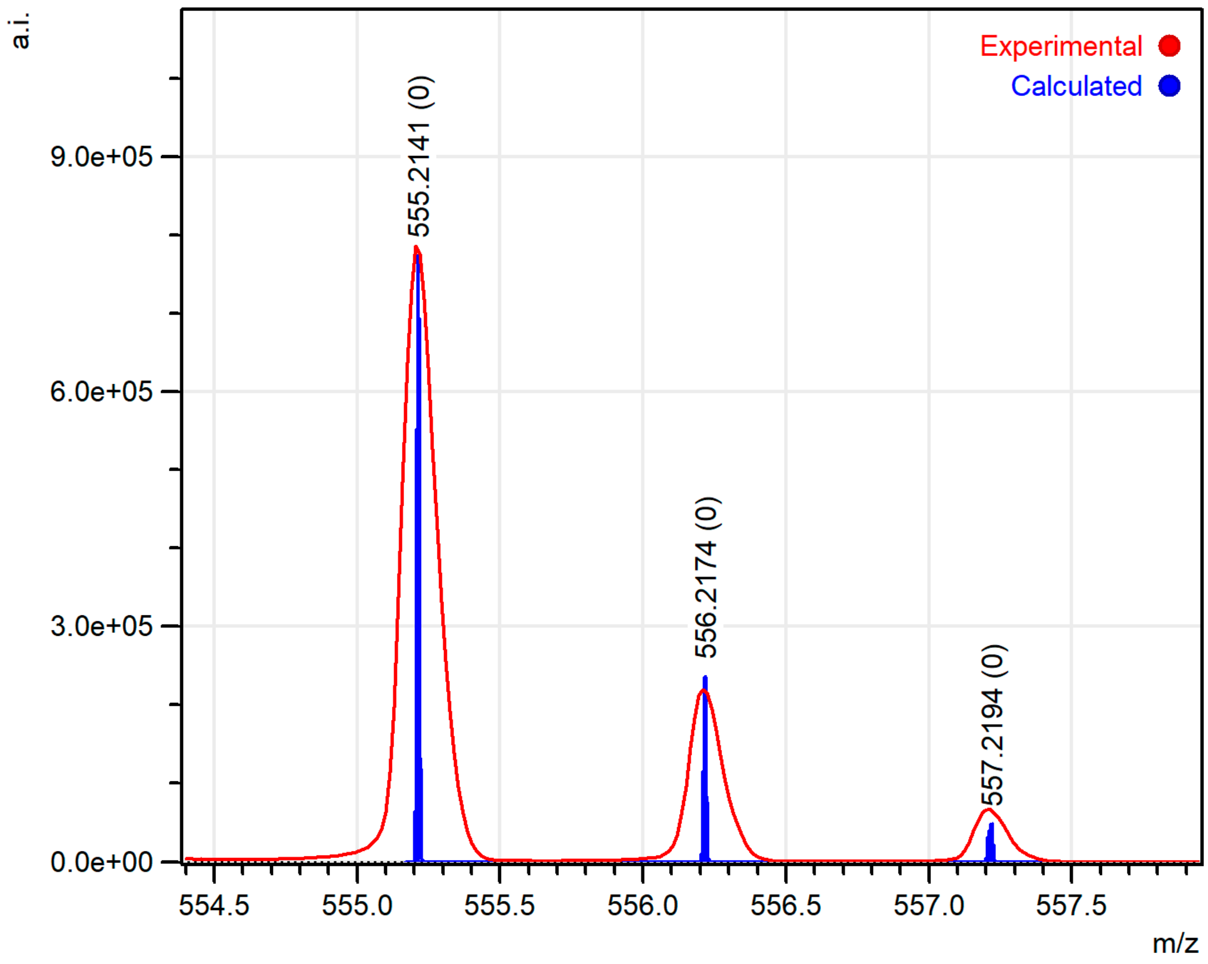

Figure S6. ESI^+ mass-spectrum of N^C ($[M+Na]^+$cation area), solvent – methanol.

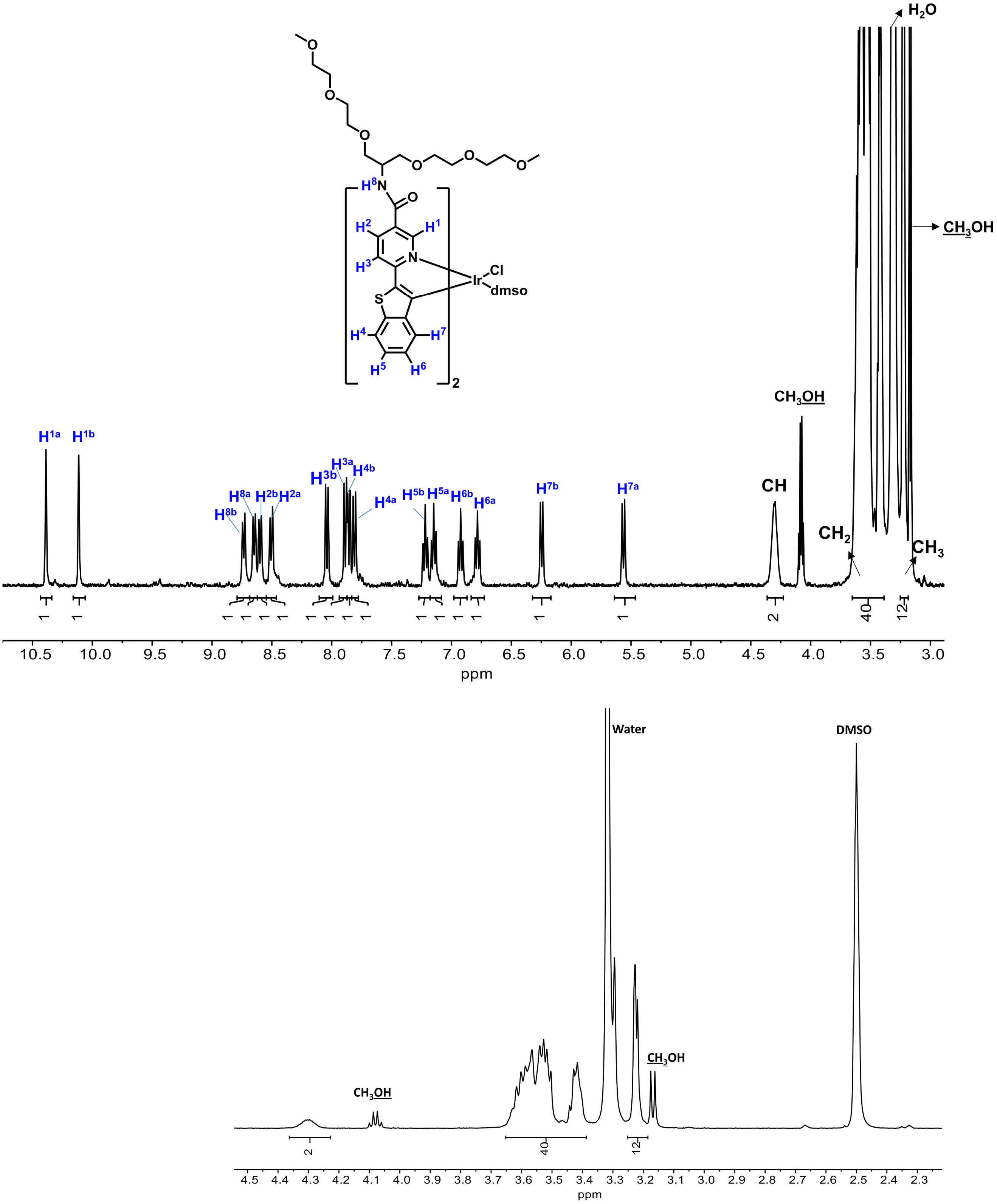

Figure S7. ^{1}H NMR spectrum of dissociated dimer [$Ir_2(N^\wedge C)_4Cl_2$], $(CD_3)_2SO$, 298 K (top – full view, bottom – aliphatic area).

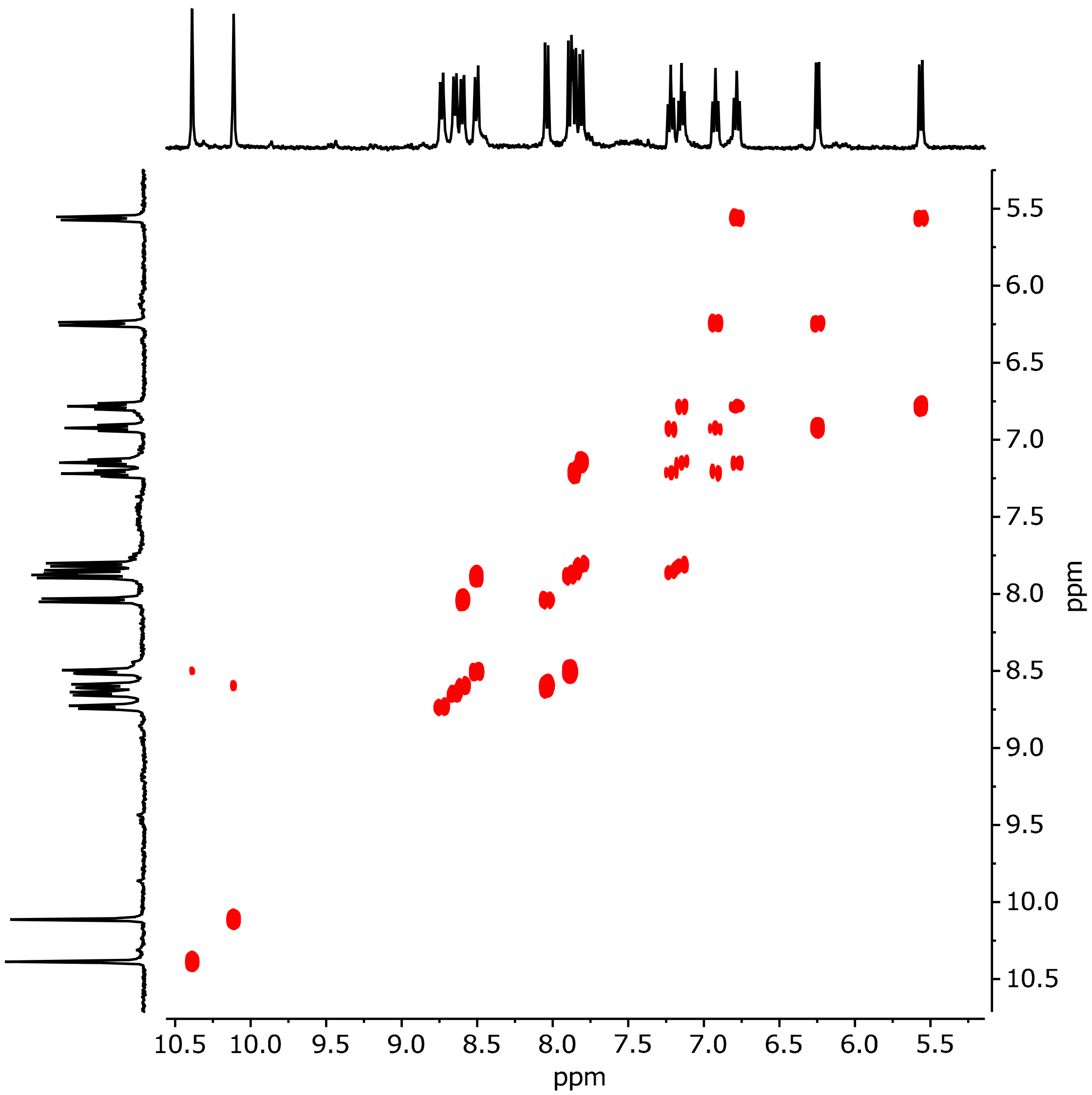

Figure S8. ^{1}H-^{1}H COSY NMR spectrum of dissociated dimer $[Ir_2(N^C)_4Cl_2]$, $(CD_3)_2SO$, 298 K.

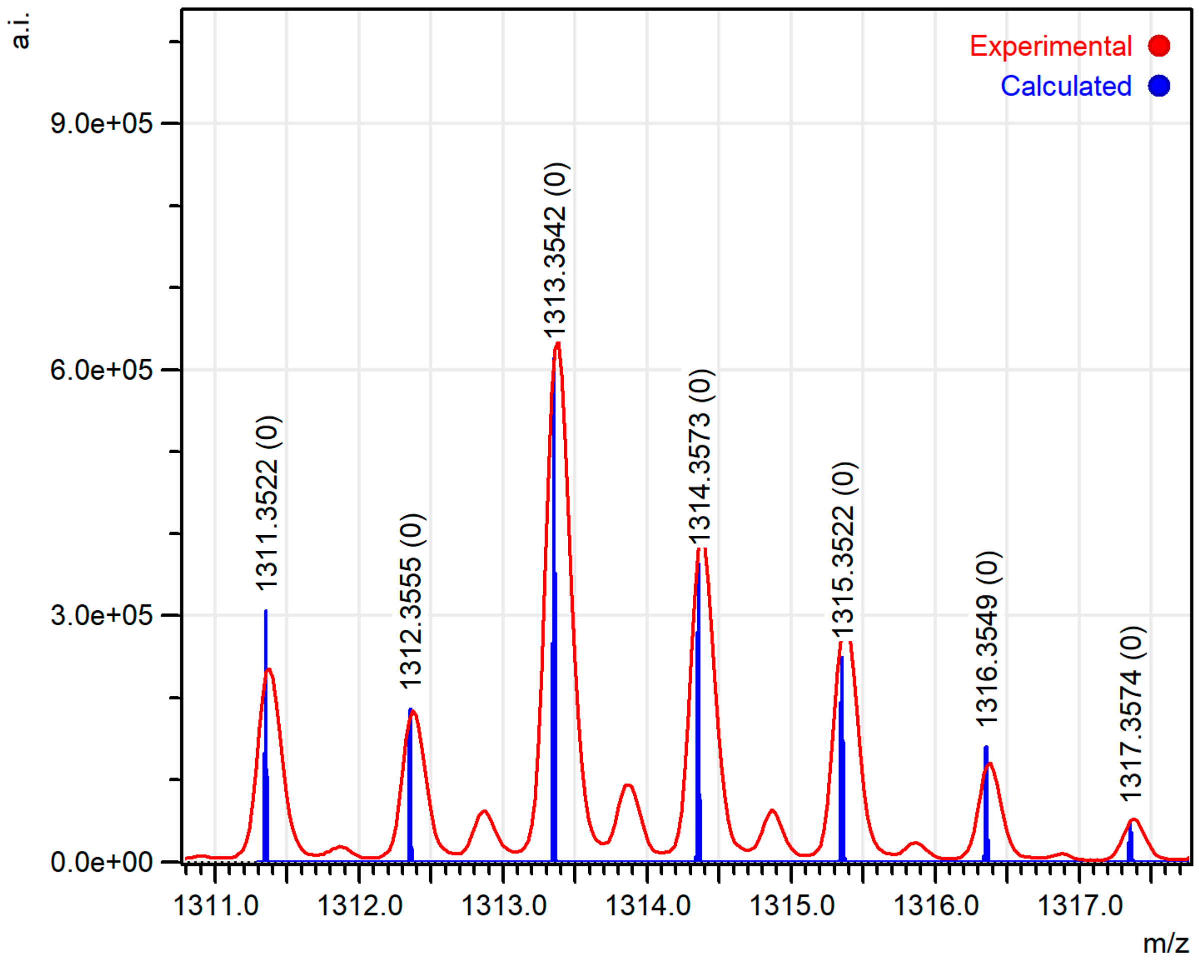

Figure S9. ESI$^+$ mass-spectrum of dissociated dimer ([Ir(N^C)$_2$Cl+Na]$^+$cation area), solvent – methanol.

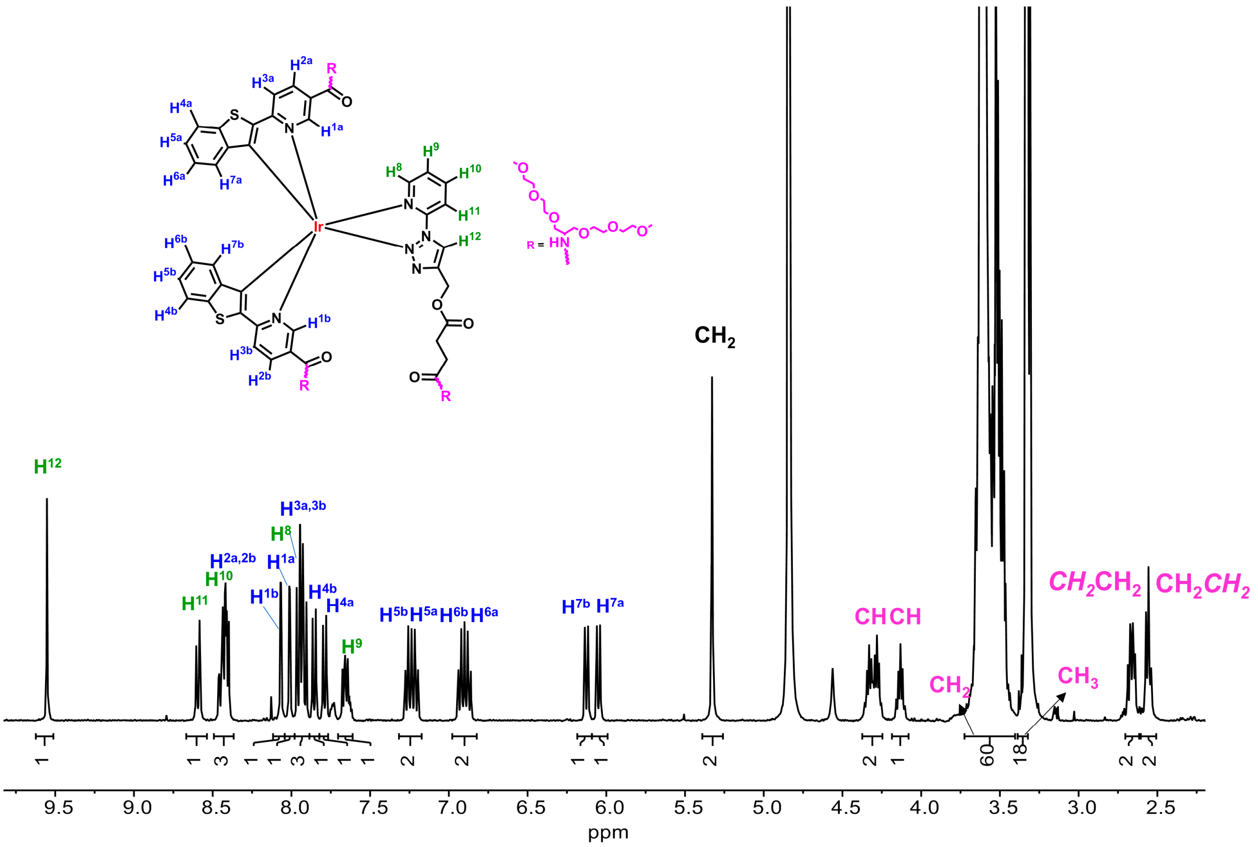

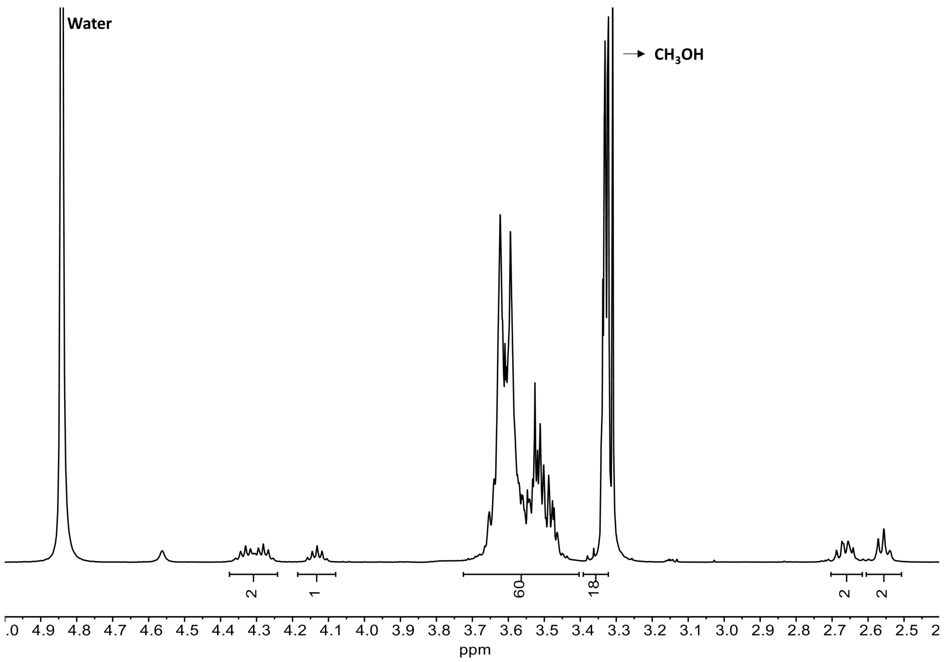

Figure S10. ^{1}H NMR spectrum of **Ir1**, CD_3OD, 298 K (top – full view, bottom – aliphatic area).

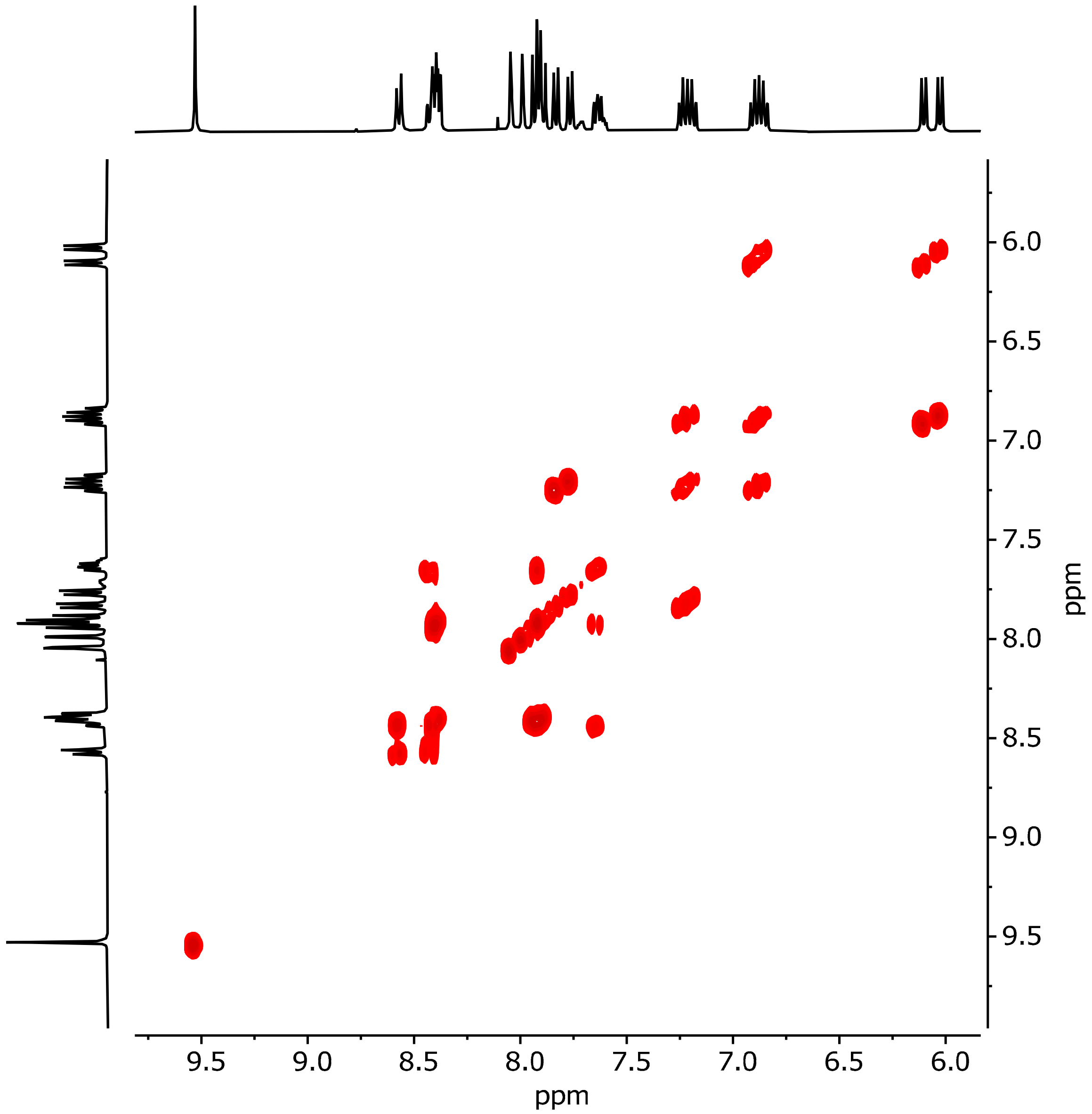

Figure S11. ^{1}H-^{1}H COSY NMR spectrum of **Ir1**, CD_3OD, 298 K.

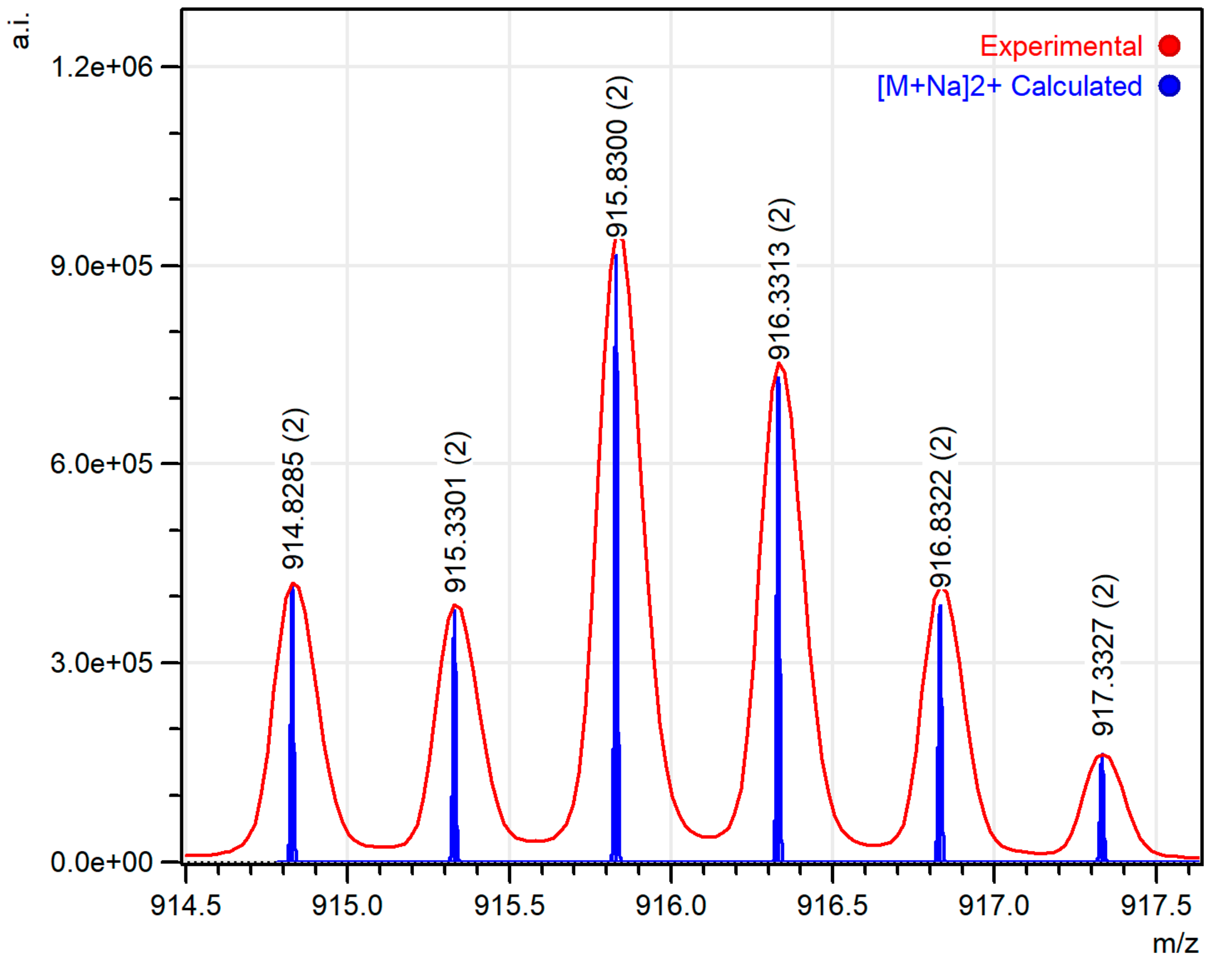

Figure S12. ESI$^+$ mass-spectrum of **Ir1** ($[M+Na]^{2+}$cation area), solvent – methanol.

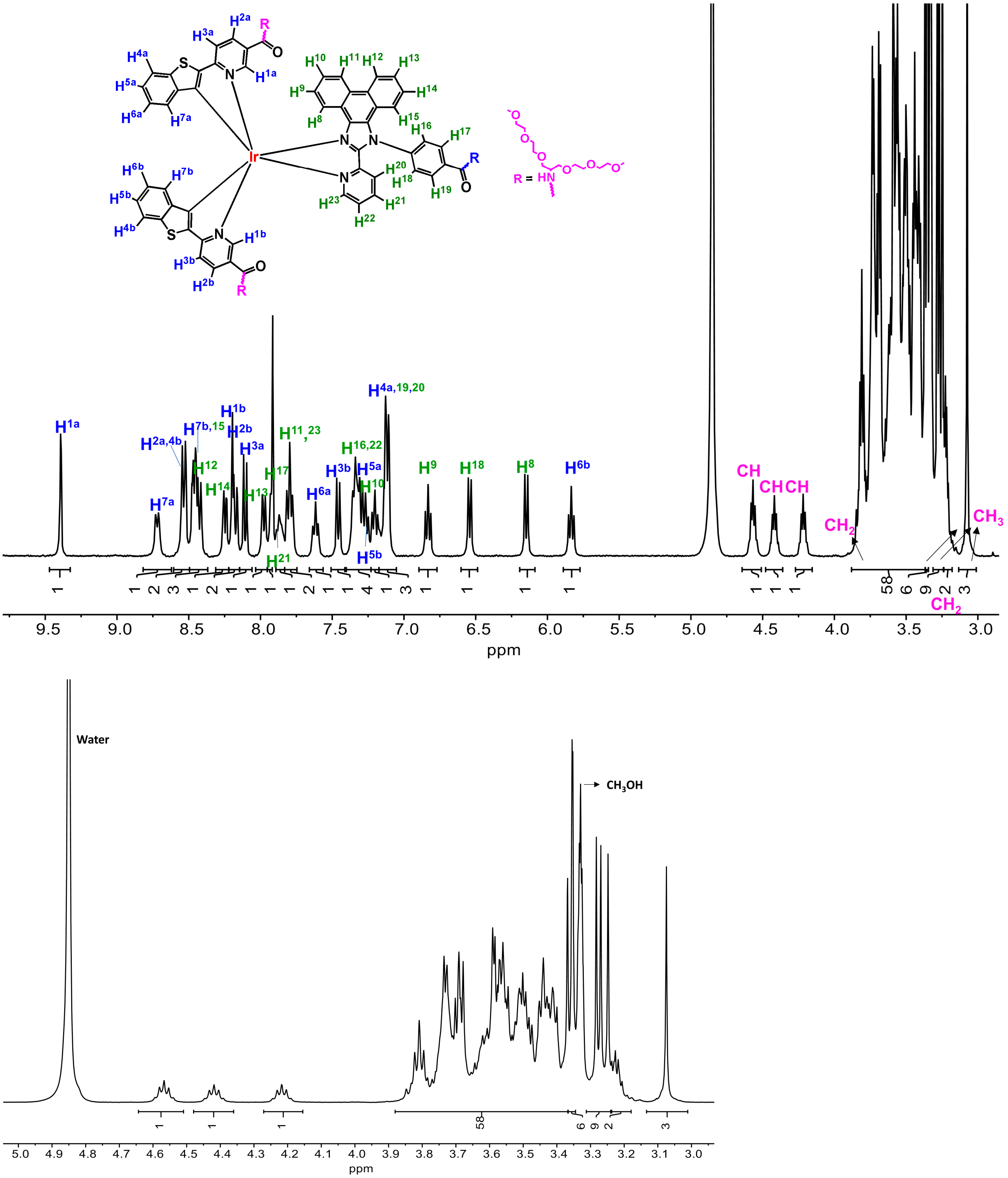

Figure S13. ^{1}H NMR spectrum of **Ir2**, CD_3OD, 298 K (top – full view, bottom – aliphatic area).

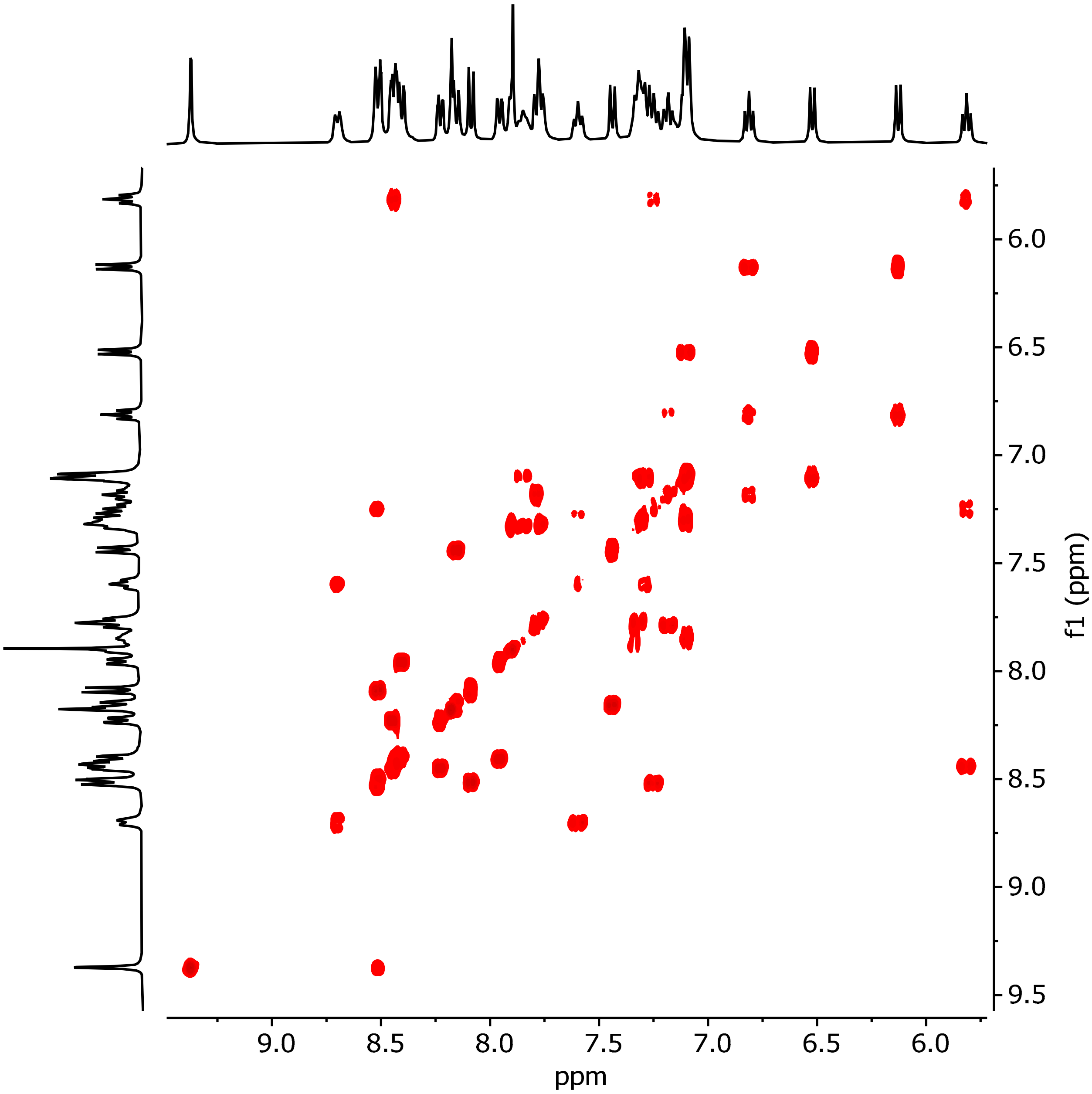

Figure S14. ^{1}H-^{1}H COSY NMR spectrum of **Ir2**, CD_3OD, 298 K.

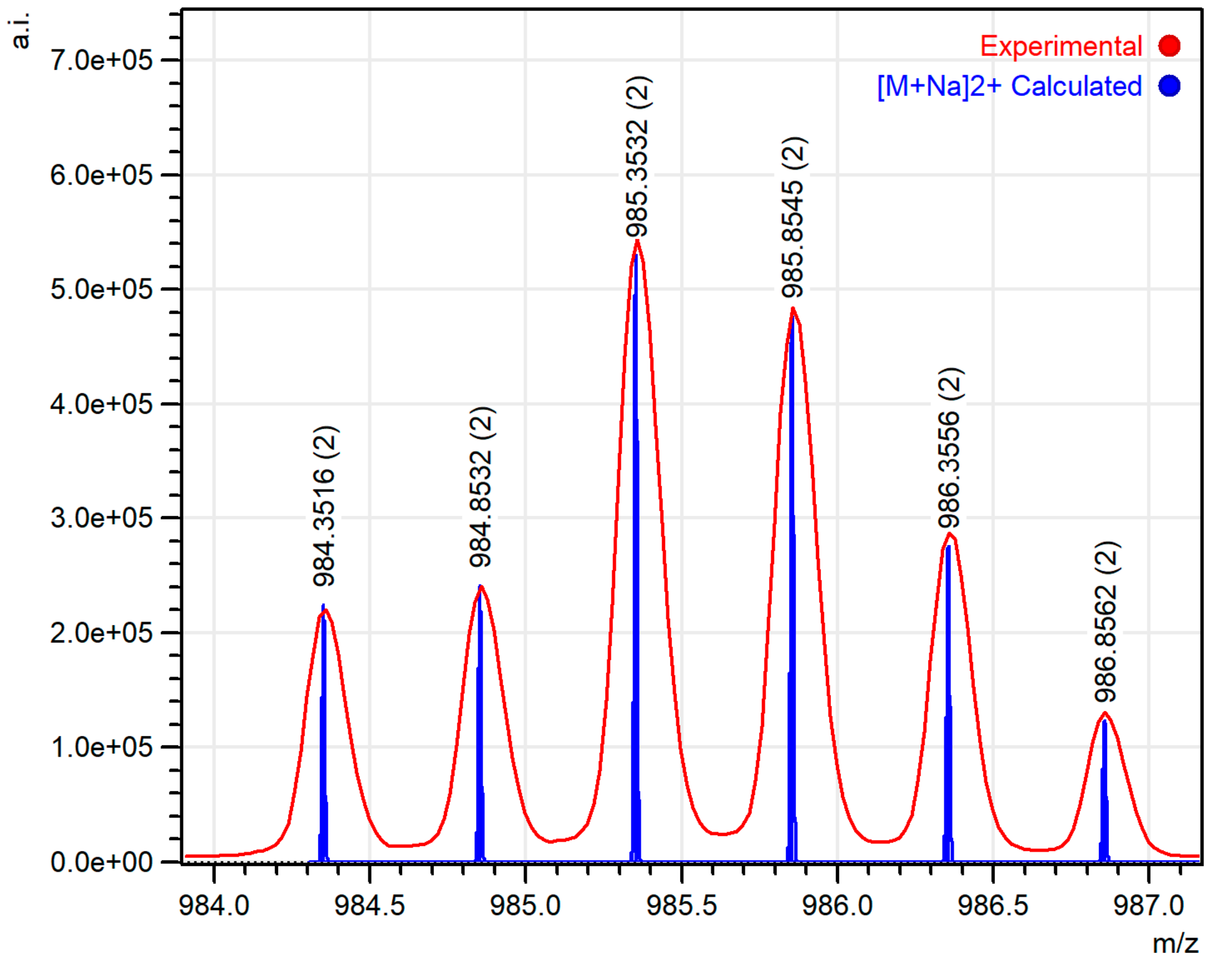

Figure S15. ESI$^+$ mass-spectrum of **Ir2** ([M+Na]$^{2+}$cation area), solvent – methanol.

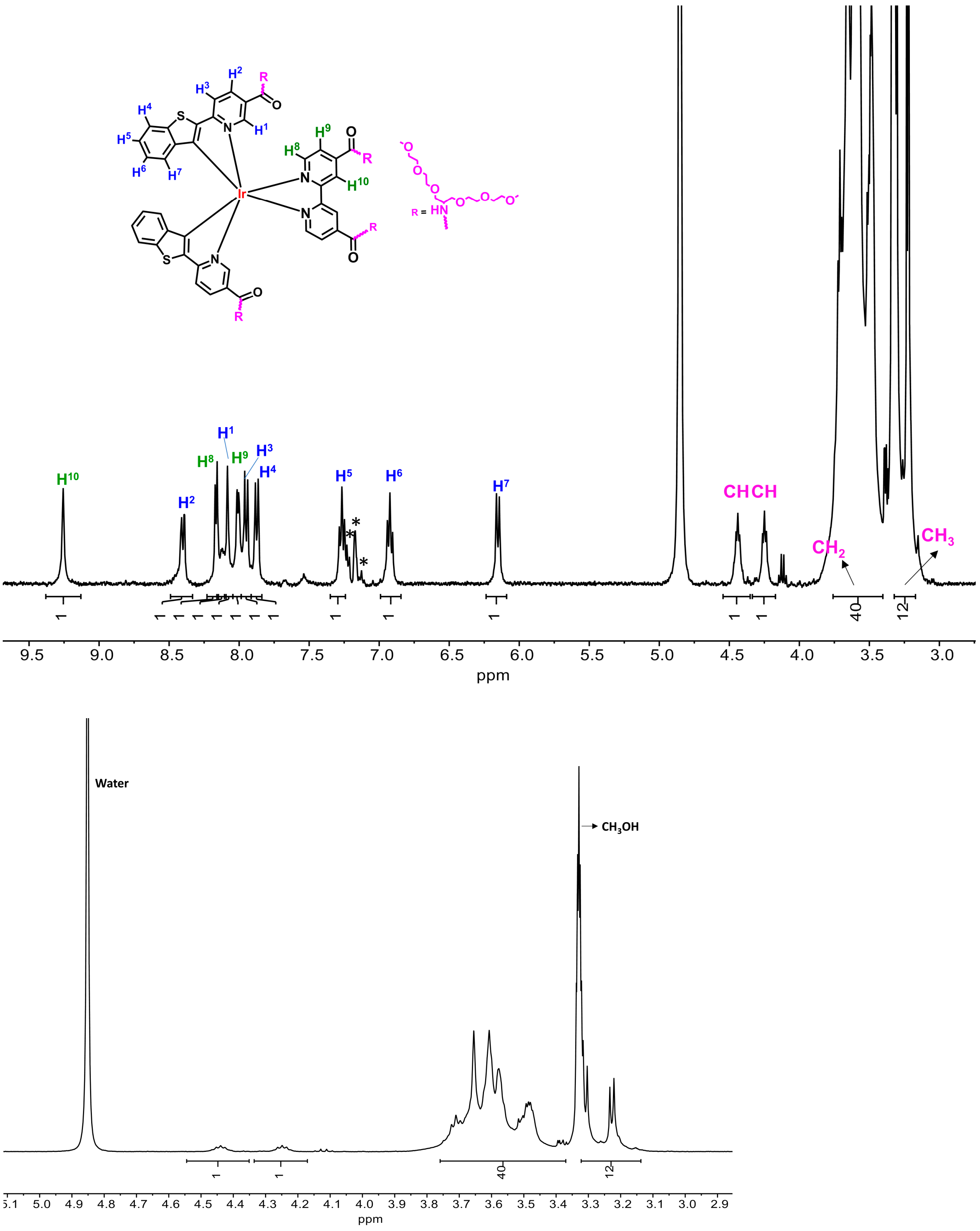

Figure S16. ^{1}H NMR spectrum of **Ir3**, CD_3OD, 298 K (top – full view, bottom – aliphatic area). Peak assignments: * -toluene in CD_3OD impurities.

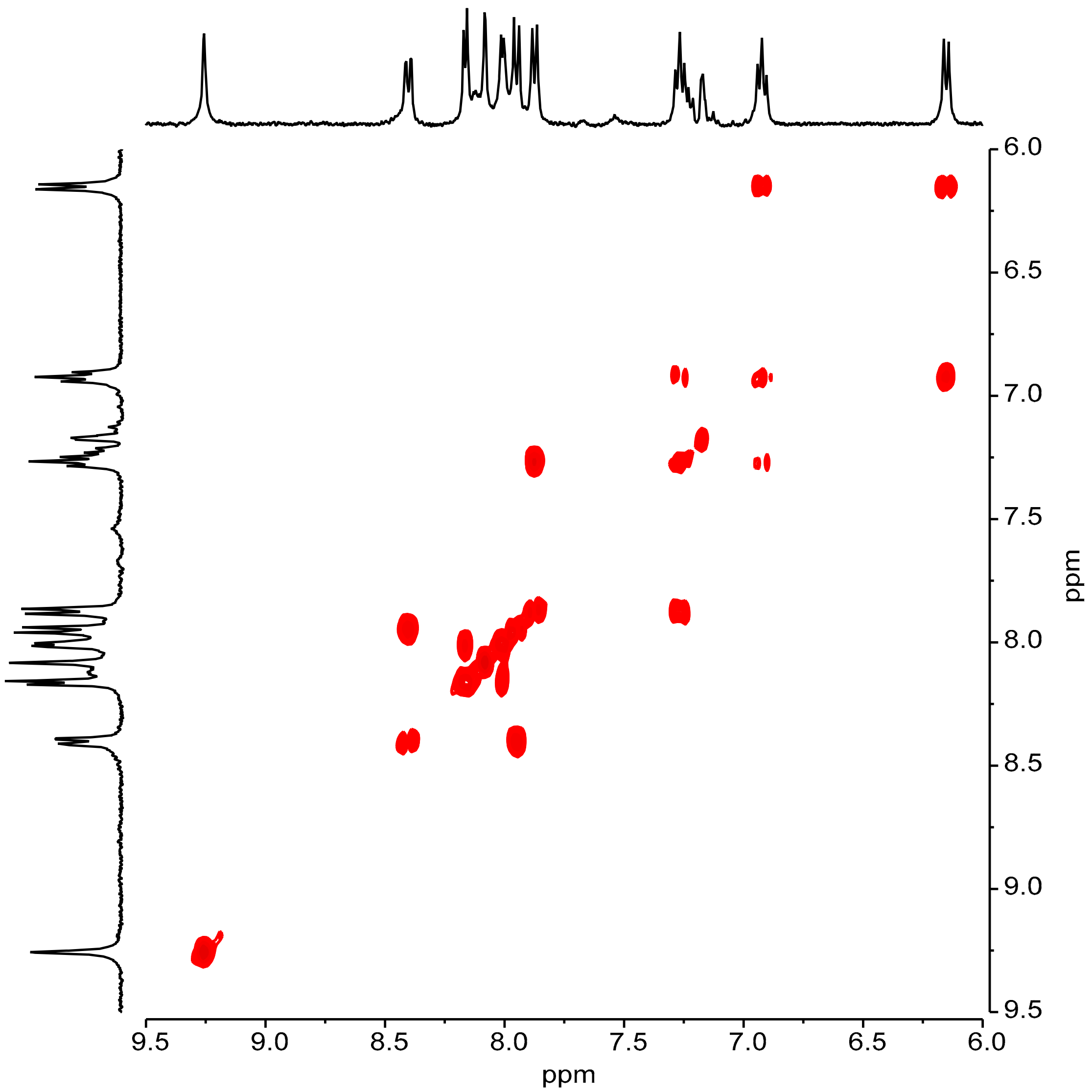

Figure S17. ^{1}H-^{1}H COSY NMR spectrum of **Ir3**, CD_3OD, 298 K.

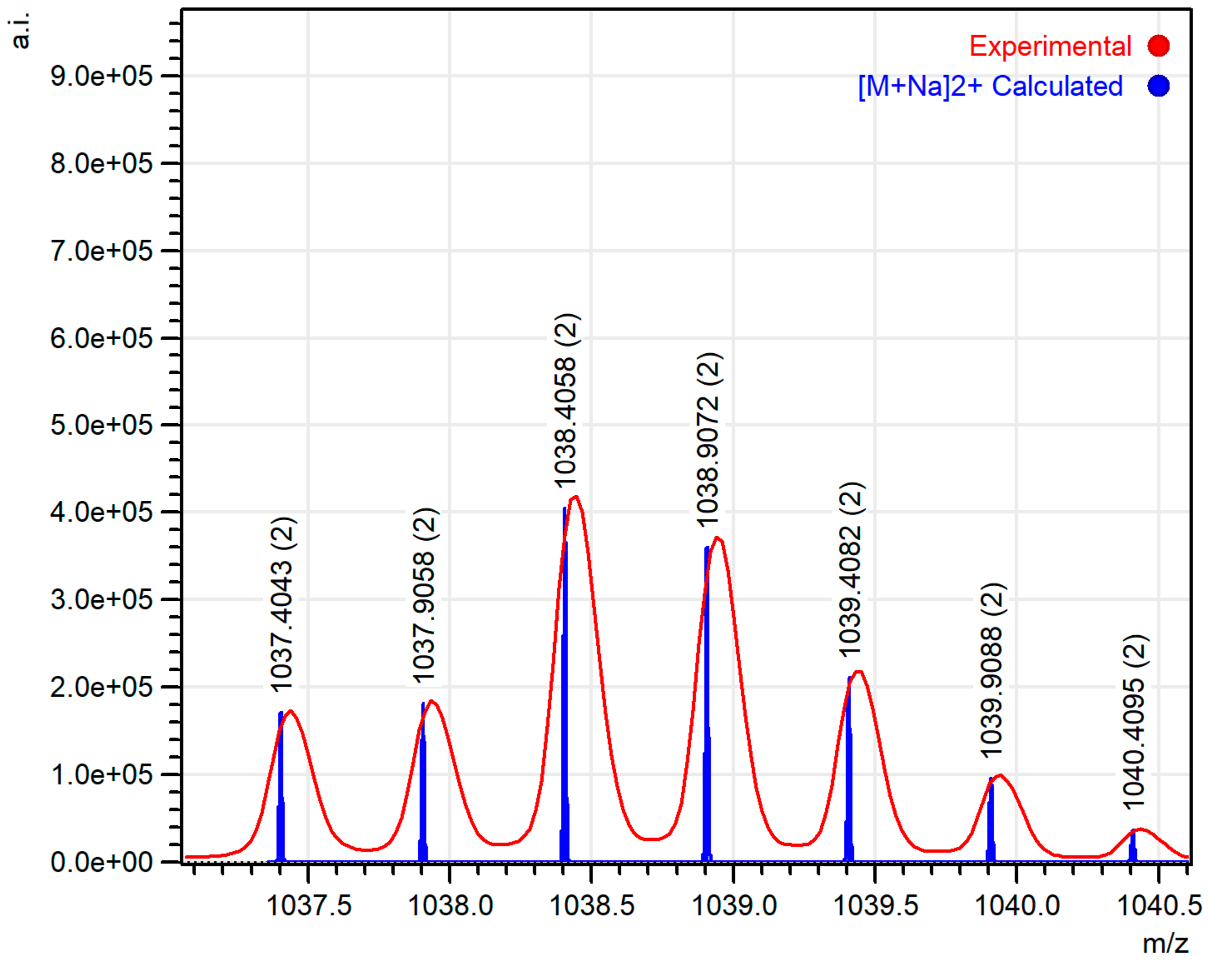

Figure S18. ESI$^+$ mass-spectrum of **Ir3** ($[M+Na]^{2+}$cation area), solvent – methanol.

4. Photophysical data

Table S1. Values of excitation state lifetime of complexes **Ir1** and **Ir2** in various aqueous media and at different oxygen concentrations. Excitation at 355 nm, T = 37°C, C(Complex) = 10 μM.

Com-plex	Water		0.01M PBS pH 7.4		DMEM + 10% FBS		0.01M PCBS pH 5.6	0.01M PCBS pH 6.5	0.01M PBS pH 8.5
	C_{O_2}, M	, μs	C_{O_2}, M	, μs		, μs	, μs (C_{O_2}, M)	, μs (C_{O_2}, M)	, μs (C_{O_2}, M)
**Ir1**	215	1.24	205	1.39	201	1.43	1.38 (207)	1.37 (206)	1.40 (205)
	169	1.50	141	1.94	128.4	2.00			
	116	2.00	92.9	2.56	82.9	2.82			
	91.1	2.44	54.7	3.69	31.8	5.44	10.11 (0.97)	10.00 (1.68)	10.09 (1.33)
	24.3	5.45	28.4	5.32	15.7	7.24			
	1.04	10.16	1.97	9.97	2.92	9.29			
**Ir2**	218	1.52	208	1.80	199	1.90	1.81 (207)	1.81 (208)	1.84 (204)
	192	1.67	164	2.11	140	2.29			
	156	1.94	121	2.55	103	2.80			
	103	2.52	108	2.70	61.5	3.42	5.20 (2.02)	5.31 (1.22)	5.30 (1.13)
	50.4	3.48	15.5	4.79	48.1	3.74			
	1.10	5.32	0.90	5.34	2.80	5.16			

PBS – phosphate buffer saline, PBCS – phosphate-citrate buffer saline.

5. Computational data

Table S2. Calculated and experimental wavelength of emission maxima of complexes **Ir1-Ir3.**

Complex	Calculated wavelength, nm	Experimental wavelength, nm
**Ir1**	625	632
**Ir2**	646	638
**Ir3**	683	655

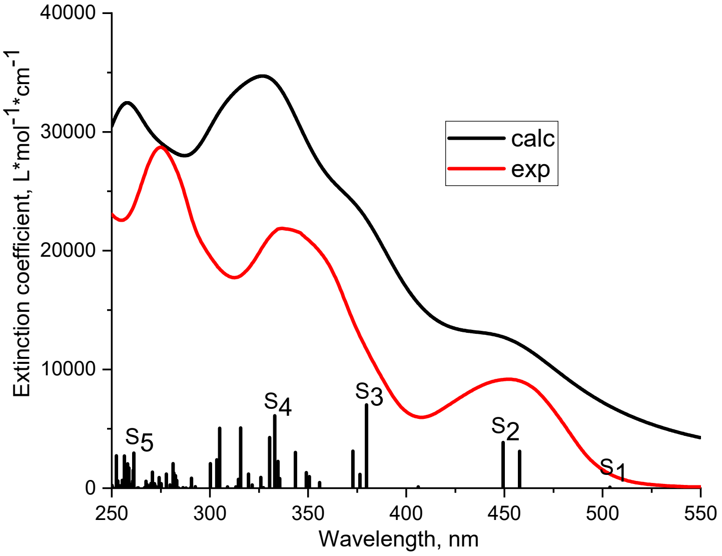

Figure S19. Absorption spectra of **Ir1**: experimental (red) and calculated (black) lines with oscillator strengths of electronic transitions (bars).

Table S3. Calculated absorption maxima (λ) and oscillator strengths (f) of complex **Ir1**.

Transitions	λ_{abs}, nm	f	Contribution of main NTO pair in transition (%)
S_0-S_5	261	0.088	66
S_0-S_4	333	0.182	90
S_0-S_3	380	0.209	95
S_0-S_2	449	0.115	96
S_0-S_1	504	0.002	97

Table S4. Natural transition orbitals (NTO) for **Ir1-0**, violet and terracotta colors show decrease and increase in electron density, respectively. The calculations have been simplified by substitution of OEG pendants of **Ir1** complex for methyl groups in **Ir1-0** structure. Atom colors: Ir–lilac; S–yellow; O–red; N–blue; C–gray, H–white. The data for the corresponding interfragment charge transfer (IFCT) are given below the figures. Diagonal values represent intraligand transitions, off-diagonal values represent a charge transfer from “Donor” to “Acceptor”.

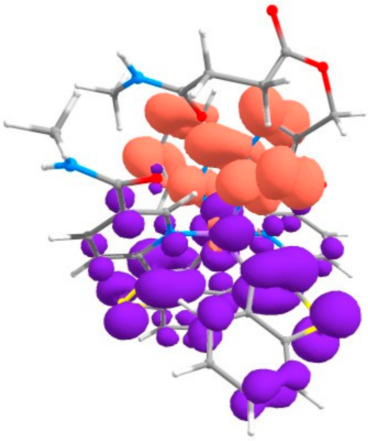

$S_0 \rightarrow S_1$

Donor	Acceptor			
	Ir	N^N	N^C	N^C′
Ir	0.005	0.177	0.006	0.002
N^N	0.000	0.011	0.000	0.000
N^C	0.011	0.408	0.014	0.004
N^C′	0.009	0.339	0.012	0.003

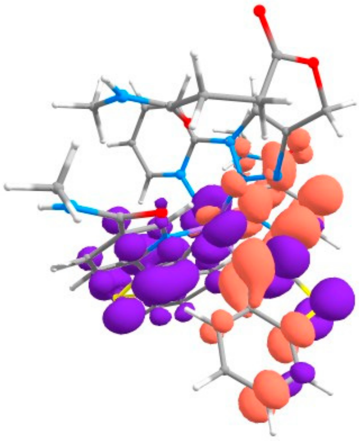

$S_0 \rightarrow S_2$

Donor	Acceptor			
	Ir	N^N	N^C	N^C′
Ir	0.007	0.006	0.176	0.001
N^N	0.000	0.000	0.011	0.000
N^C	0.015	0.013	0.407	0.001
N^C′	0.012	0.011	0.338	0.001

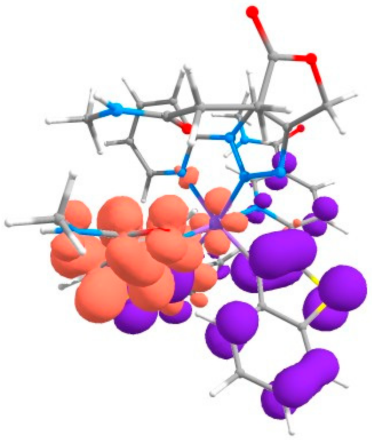

$S_0 \rightarrow S_3$

Donor	Acceptor			
	Ir	N^N	N^C	N^C′
Ir	0.000	0.000	0.000	0.004
N^N	0.000	0.000	0.000	0.005

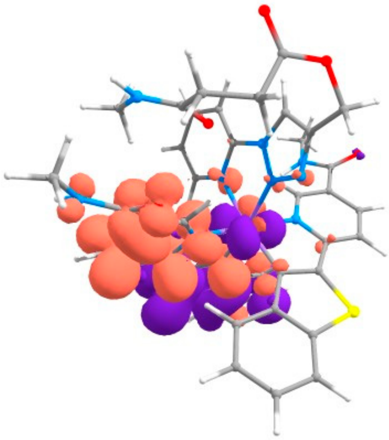

$S_0 \rightarrow S_4$

Donor	Acceptor			
	Ir	N^N	N^C	N^C′
Ir	0.005	0.010	0.011	0.129
N^N	0.001	0.001	0.002	0.018

N^C	0.015	0.010	0.022	0.414
N^C′	0.018	0.011	0.026	0.474

N^C	0.002	0.004	0.005	0.051
N^C′	0.026	0.050	0.055	0.629

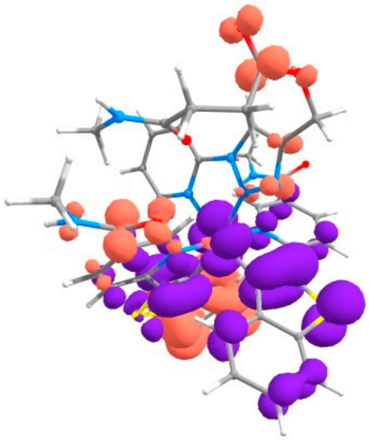

$S_0 \rightarrow S_5$

Donor	Acceptor			
	Ir	N^N	N^C	N^C′
Ir	0.006	0.020	0.040	0.169
N^N	0.001	0.003	0.007	0.028
N^C	0.008	0.030	0.061	0.257
N^C′	0.009	0.031	0.064	0.267

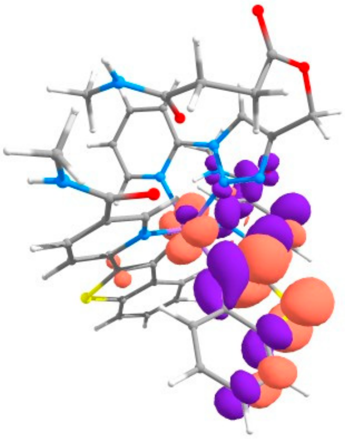

$T_1 \rightarrow S_0$

Donor	Acceptor			
	Ir	N^N	N^C	N^C′
Ir	0.004	0.000	0.030	0.001
N^N	0.002	0.000	0.013	0.000
N^C	0.104	0.006	0.813	0.023
N^C′	0.000	0.000	0.003	0.000

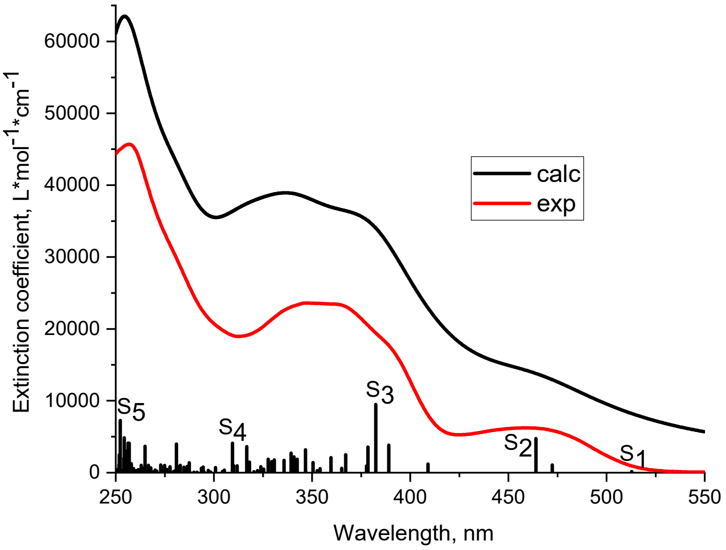

Figure S20. Absorption spectra of **Ir2**: experimental (red) and calculated (black) lines with oscillator strengths of electronic transitions (bars).

Table S5. Calculated absorption maxima (λ) and oscillator strengths (f) of complex **Ir2**.

Transitions	λ_{abs}, nm	*f*	Contribution of main NTO pair in transition (%)
S_0-S_5	254	0.145	32
S_0-S_4	309	0.122	47
S_0-S_3	382	0.283	84
S_0-S_2	464	0.141	93
S_0-S_1	513	0.004	95

Table S6. Natural transition orbitals (NTO) for **Ir2-0**, violet and terracotta colors show decrease and increase in electron density, respectively. The calculations have been simplified by substitution of OEG pendants of **Ir2** complex for methyl groups in **Ir2-0** structure. Atom colors: Ir–lilac; S–yellow; O–red; N–blue; C–gray, H–white. The data for the corresponding interfragment charge transfer (IFCT) are given below the figures. Diagonal values represent intraligand transitions, off-diagonal values represent a charge transfer from “Donor” to “Acceptor”.

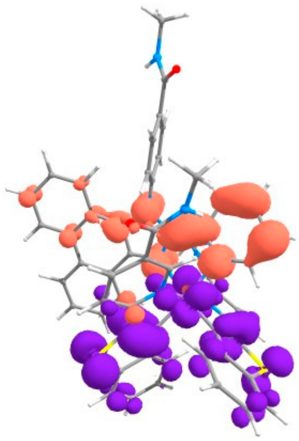

$S_0 \rightarrow S_1$

Donor	Acceptor			
	Ir	N^N	N^C	N^C′
Ir	0.002	0.177	0.024	0.004
N^N	0.000	0.031	0.004	0.001
N^C	0.003	0.376	0.052	0.008
N^C′	0.002	0.272	0.037	0.006

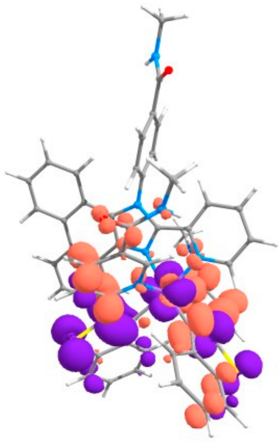

$S_0 \rightarrow S_2$

Donor	Acceptor			
	Ir	N^N	N^C	N^C′
Ir	0.008	0.006	0.065	0.129
N^N	0.001	0.001	0.011	0.023
N^C	0.016	0.012	0.137	0.273
N^C′	0.012	0.009	0.099	0.198

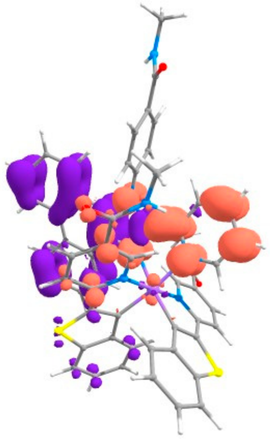

$S_0 \rightarrow S_3$

Donor	Acceptor			
	Ir	N^N	N^C	N^C′
Ir	0.000	0.013	0.002	0.001

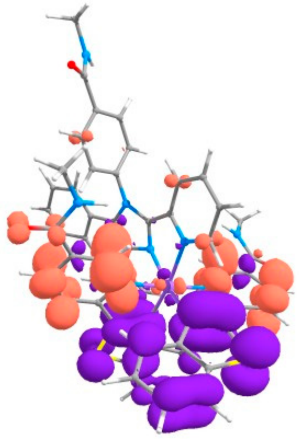

$S_0 \rightarrow S_4$

Donor	Acceptor			
	Ir	N^N	N^C	N^C′
Ir	0.003	0.017	0.039	0.051

N^N	0.007	0.652	0.105	0.041
N^C	0.001	0.103	0.017	0.007
N^C′	0.000	0.042	0.007	0.003

N^N	0.005	0.032	0.075	0.095
N^C	0.006	0.039	0.090	0.114
N^C′	0.010	0.068	0.157	0.198

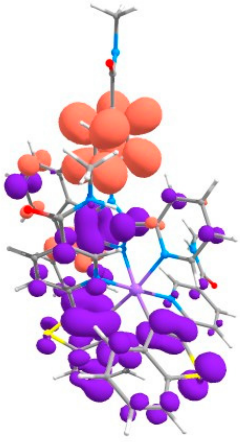

$S_0 \rightarrow S_5$

Donor	Acceptor			
	Ir	N^N	N^C	N^C′
Ir	0.001	0.068	0.005	0.007
N^N	0.003	0.378	0.027	0.039
N^C	0.002	0.235	0.017	0.024
N^C′	0.001	0.163	0.012	0.017

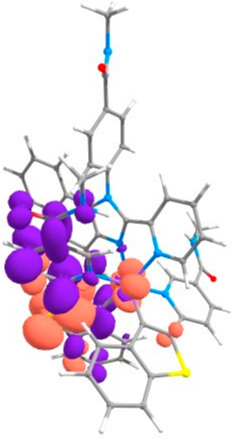

$T_1 \rightarrow S_0$

Donor	Acceptor			
	Ir	N^N	N^C	N^C′
Ir	0.005	0.002	0.030	0.001
N^N	0.006	0.002	0.037	0.001
N^C	0.113	0.047	0.709	0.026
N^C′	0.003	0.001	0.017	0.001

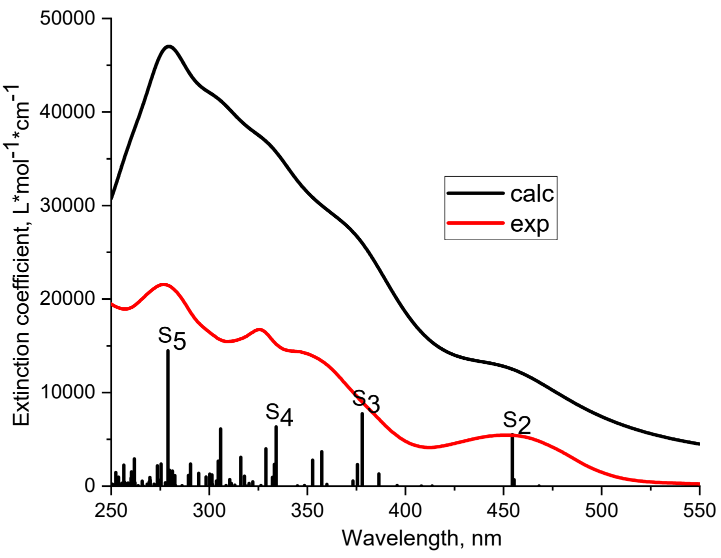

Figure S21. Absorption spectra of **Ir3**: experimental (red) and calculated (black) lines with oscillator strengths of electronic transitions (bars).

Table S7. Calculated absorption maxima (λ) and oscillator strengths (f) of complex **Ir3**.

Transitions	$λ_{abs}$, nm	*f*	Contribution of main NTO pair in transition (%)
S_0-S_5	279	0.432	42
S_0-S_4	334	0.189	88
S_0-S_3	378	0.231	92
S_0-S_2	455	0.164	96
S_0-S_1	601	0.002	98

Table S8. Natural transition orbitals (NTO) for **Ir3-0**, violet and terracotta colors show decrease and increase in electron density, respectively. The calculations have been simplified by substitution of OEG pendants of **Ir3** complex for methyl groups in **Ir3-0** structure. Atom colors: Ir–lilac; S–yellow; O–red; N–blue; C–gray, H–white. The data for the corresponding interfragment charge transfer (IFCT) are given below the figures. Diagonal values represent intraligand transitions, off-diagonal values represent a charge transfer from "Donor" to "Acceptor".

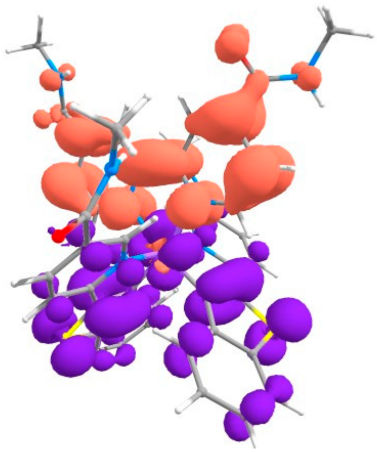

$S_0 \rightarrow S_1$

Donor	Acceptor			
	Ir	N^N	N^C	N^C′
Ir	0.004	0.195	0.002	0.002
N^N	0.000	0.010	0.000	0.000
N^C	0.007	0.379	0.005	0.004
N^C′	0.007	0.378	0.005	0.004

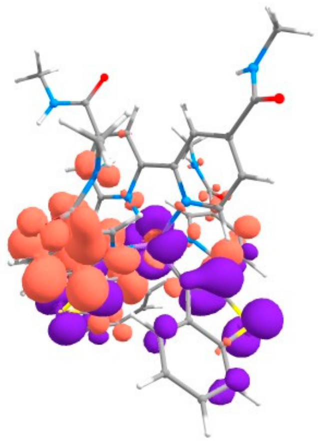

$S_0 \rightarrow S_2$

Donor	Acceptor			
	Ir	N^N	N^C	N^C′
Ir	0.007	0.008	0.055	0.133
N^N	0.000	0.000	0.003	0.007
N^C	0.013	0.016	0.106	0.259
N^C′	0.013	0.016	0.106	0.258

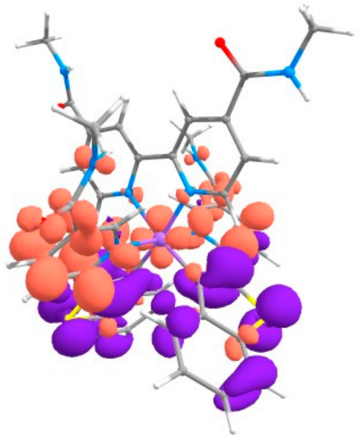

$S_0 \rightarrow S_3$

Donor	Acceptor			
	Ir	N^N	N^C	N^C′
Ir	0.000	0.000	0.002	0.002

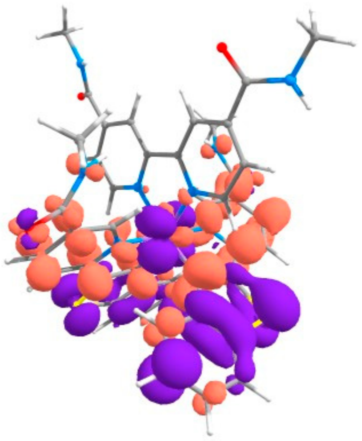

$S_0 \rightarrow S_4$

Donor	Acceptor			
	Ir	N^N	N^C	N^C′
Ir	0.008	0.007	0.072	0.117

N^N	0.000	0.000	0.002	0.002
N^C	0.018	0.026	0.227	0.215
N^C′	0.019	0.027	0.236	0.223

N^N	0.001	0.001	0.011	0.018
N^C	0.011	0.010	0.106	0.174
N^C′	0.017	0.015	0.164	0.267

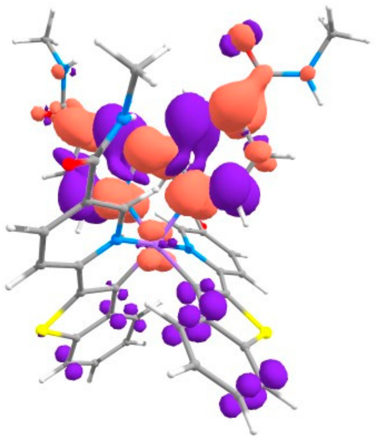

$S_0 \rightarrow S_5$

Donor	Acceptor			
	Ir	N^N	N^C	N^C′
Ir	0.004	0.278	0.011	0.011
N^N	0.006	0.409	0.017	0.016
N^C	0.002	0.107	0.004	0.004
N^C′	0.002	0.119	0.005	0.005

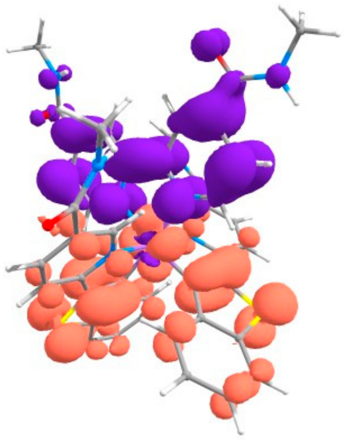

$T_1 \rightarrow S_0$

Donor	Acceptor			
	Ir	N^N	N^C	N^C′
Ir	0.004	0.000	0.009	0.009
N^N	0.183	0.009	0.385	0.381
N^C	0.002	0.000	0.004	0.004
N^C′	0.002	0.000	0.004	0.004

6. Optimized structures data

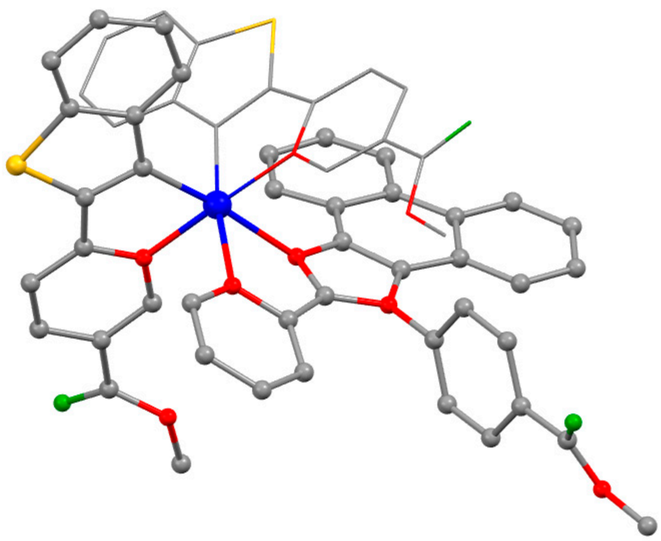

Figure S22. Optimized structure of the model **Ir2-0** complex (hydrogen atoms are omitted for clarity). The calculations have been simplified by substitution of OEG pendants in **Ir2** complex for methyl groups in **Ir2-0** structure. Atom colors: Ir–blue; S–yellow; O–green; N–red; C–gray.

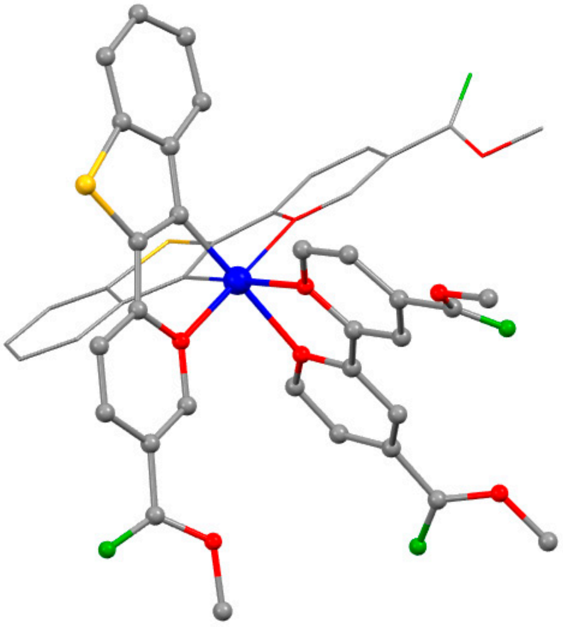

Figure S23. Optimized structure of the model **Ir3-0** complex (hydrogen atoms are omitted for clarity). The calculations have been simplified by substitution of OEG pendants in **Ir3** complex for methyl groups in **Ir3-0** structure. Atom colors: Ir–blue; S–yellow; O–green; N–red; C–gray.

7. Biological experiments data.

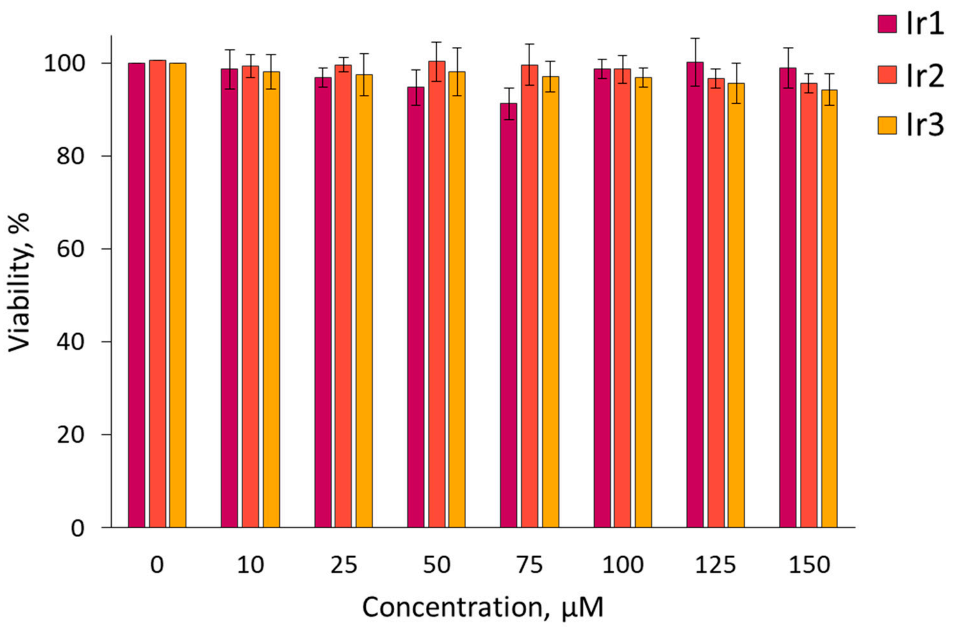

Figure S24. MTT-assay for viability of HCT116 cells after incubation with complexes **Ir1-Ir3**. Mean ± SD. N = 3 repeats by 10 wells.

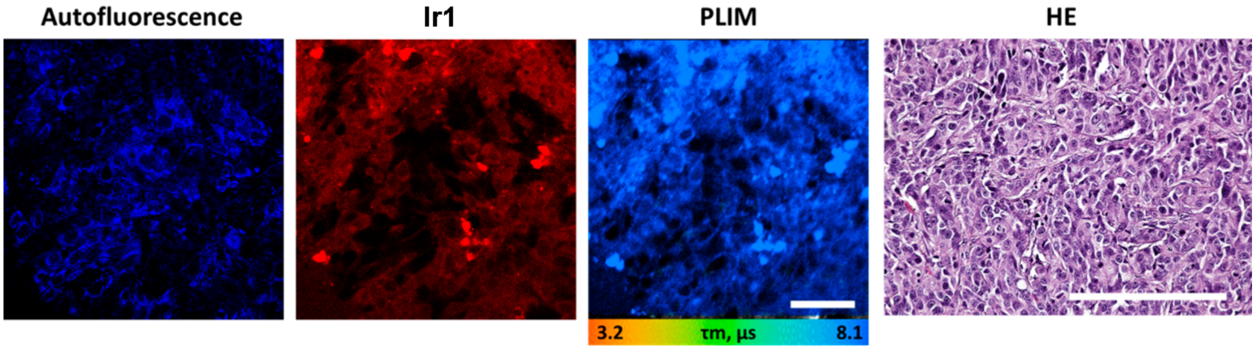

Figure S25. *In vivo* PLIM of human colorectal tumor HCT116 with the **Ir1** complex. Representative microscopic images of cellular autofluorescence, **Ir1** luminescence intensity, PLIM (Scale bar = 50 µm.) and Histological verification (Scale bar = 150 µm).

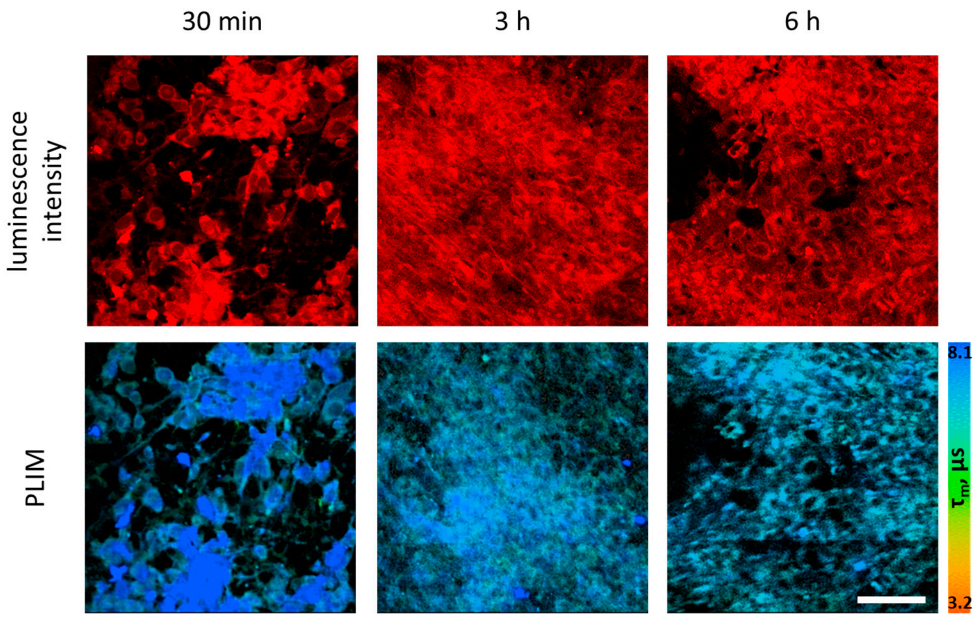

Figure S26. *In vivo* PLIM of mouse colorectal tumor CT26 with the **Ir1** complex. Representative microscopic images of **Ir1** luminescence intensity, PLIM at 30 minutes, 3 hours and 6 hours after sensor injection. Scale bar = 50 μm.

Table S9. The key structural parameters of complex **Ir1-0** (R = NHMe) and its literature analog[10].

Complex **Ir1**	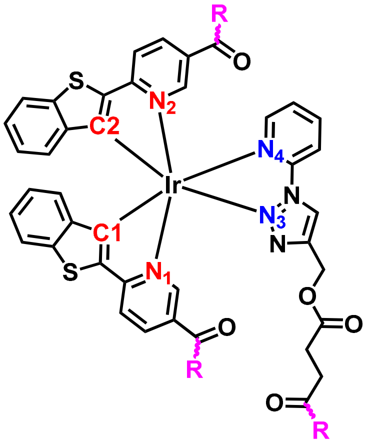	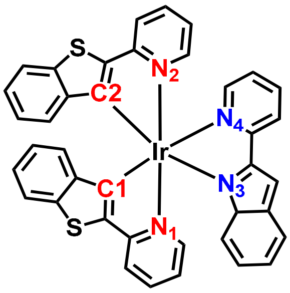
Distances, A°		
Ir-C1	2.006	2.040(6)
Ir-N1	2.076	2.074(5)
Ir-C2	2.002	2.049(6)
Ir-N2	2.068	2.070(5)
Ir-N3	2.116	2.093(5)
Ir-N4	2.152	2.130(5)
Angles, °		
N1-Ir-C1	80.59	80.1(2)
N2-Ir-C2	80.59	79.7(2)
N3-Ir-N4	76.05	77.52(19)
C1-Ir-C2	91.12	88.8(2)
C2-Ir-N1	99.13	100.0(2)
C2-Ir-N4	96.51	94.8(2)
C1-Ir-N2	98.73	97.4(2)
N2-Ir-N4	86.54	90.14(18)
N4-Ir-N1	94.18	92.4(2)
N3-Ir-N2	96.47	92.09(19)
N3-Ir-N1	83.89	88.54(19)
N3-Ir-C1	96.47	99.9(2)
N3-Ir-C2	172.21	168.8(2)
N2-Ir-N1	179.26	177.49(19)
C1-Ir-N4	171.37	172.1(2)

Table S10. The key structural parameters of complex **Ir2-0** (R = NHMe) and its literature analog[11].

Complex **Ir2**	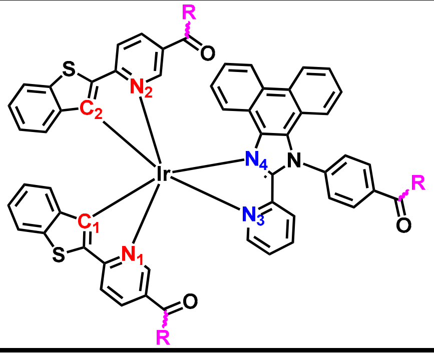	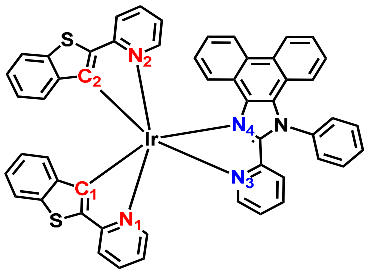
Distances, A°		
Ir-C1	1.997	2.014(10)
Ir-N1	2.062	2.043(8)
Ir-C2	2.008	2.014(10)
Ir-N2	2.080	2.069(8)
Ir-N3	2.146	2.147(9)
Ir-N4	2.180	2.199(8)
Angles, °		
N1-Ir-C1	80.54	80.3(4)
N2-Ir-C2	80.05	79.9(4)
N3-Ir-N4	76.36	76.2(3)
C1-Ir-C2	88.11	85.3(4)
C2-Ir-N1	100.55	98.9(4)
C2-Ir-N4	98.82	102.3(3)
C1-Ir-N2	100.95	96.5(4)
N2-Ir-N4	81.54	87.4(3)
N4-Ir-N1	96.93	95.9(3)
N3-Ir-N2	93.00	95.5(3)
N3-Ir-N1	86.30	85.8(3)
N3-Ir-C1	96.88	96.3(4)
N3-Ir-C2	172.14	175.2(4)
N2-Ir-N1	178.43	176.7(4)
C1-Ir-N4	172.97	172.0(4)

Table S11. The key structural parameters of complex **Ir3-0** (R = NHMe) and its literature analog[12].

Complex **Ir3**	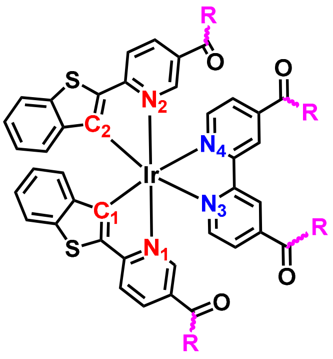	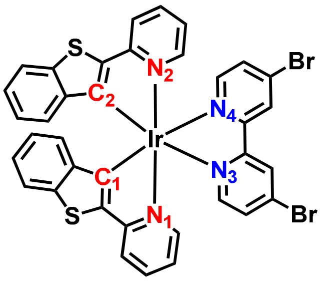
Distances, A°		
Ir-C1	2.010	2.013(5)
Ir-N1	2.073	2.052(4)
Ir-C2	2.010	2.018(5)
Ir-N2	2.073	2.055(4)
Ir-N3	2.130	2.122(4)
Ir-N4	2.131	2.112(4)
Angles, °		
N1-Ir-C1	80.41	80.48(19)
N2-Ir-C2	80.39	79.98(18)
N3-Ir-N4	77.24	76.89(16)
C1-Ir-C2	89.59	87.3(2)
C2-Ir-N1	99.73	98.30(18)
C2-Ir-N4	96.58	96.80(18)
C1-Ir-N2	99.66	96.29(18)
N2-Ir-N4	85.06	86.06(15)
N4-Ir-N1	94.86	97.26(16)
N3-Ir-N2	94.24	95.62(16)
N3-Ir-N1	85.64	86.41(16)
N3-Ir-C1	96.90	99.09(18)
N3-Ir-C2	172.22	172.62(18)
N2-Ir-N1	179.87	176.44(16)
C1-Ir-N4	172.81	175.55(18)

References

[1] Frisch, M.J.; Trucks, G.W.; Schlegel, H.B.; Scuseria, G.E.; Robb, M.A.; Cheeseman, J.R.; Scalmani, G.; Barone, V.; Petersson, G.A.; Nakatsuji, X.; et al. Gaussian 16, Revision B.01, (2016). Available online: https://gaussian.com/citation/ (accessed on 1 June 2023).

[2] Austin, A.; Petersson, G.A.; Frisch, M.J.; Dobek, F.J.; Scalmani, G.; Throssell, K. A Density Functional with Spherical Atom Dispersion Terms. *J. Chem. Theory Comput.* **2012**, *8*, 4989–5007. https://doi.org/10.1021/ct300778e.

[3] Dolg, M.; Wedig, U.; Stoll, H.; Preuss, H. Energy-adjustedabinitiopseudopotentials for the first row transition elements. *J. Chem. Phys.* **1987**, *86*, 866–872. https://doi.org/10.1063/1.452288.

[4] Tomasi, J.; Mennucci, B.; Cammi, R. Quantum Mechanical Continuum Solvation Models. *Chem. Rev.* **2005**, *105*, 2999–3094. https://doi.org/10.1021/cr9904009.

[5] O'Boyle, N.M.; Tenderholt, A.L.; Langner, K.M. cclib: A library for package-independent computational chemistry algo-rithms. *J. Comput. Chem.* **2008**, *29*, 839–845. https://doi.org/10.1002/jcc.20823.

[6] Martin, R.L. Natural transition orbitals. *J. Chem. Phys.* **2003**, *118*, 4775–4777. https://doi.org/10.1063/1.1558471.

[7] Lu, T.; Chen, F. Multiwfn: A multifunctional wavefunction analyzer. *J. Comput. Chem.* **2012**, *33*, 580–592. https://doi.org/10.1002/jcc.22885.

[8] Conway, J.; Warren, S.C.; Herrmann, D.; Murphy, K.J.; Cazet, A.; Vennin, C.; Shearer, R.F.; Killen, M.; Magenau, A.; Mélé-nec, P.; et al. Intravital Imaging to Monitor Therapeutic Response in Moving Hypoxic Regions Resistant to PI3K Pathway Targeting in Pancreatic Cancer. *Cell Rep.* **2018**, *23*, 3312–3326. https://doi.org/10.1016/j.celrep.2018.05.038.

[9] Parshina, Y.P.; Komarova, A.D.; Bochkarev, L.N.; Kovylina, T.A.; Plekhanov, A.A.; Klapshina, L.G.; Konev, A.N.; Mozherov, A.M.; Shchechkin, I.D.; Sirotkina, M.A.; et al. Simultaneous Probing of Metabolism and Oxygenation of Tumors In Vivo Using FLIM of NAD(P)H and PLIM of a New Polymeric Ir(III) Oxygen Sensor. *Int. J. Mol. Sci.* **2022**, *23*, 10263. https://doi.org/10.3390/ijms231810263.

[10] Lai, P.-N.; Teets, T.S. Ancillary Ligand Effects on Red-Emitting Cyclometalated Iridium Complexes. *Chem.—A Eur. J.* **2019**, *25*, 6026–6037. https://doi.org/10.1002/chem.201900829

[11] Solomatina, A.I.; Kuznetsov, K.M.; Gurzhiy, V.V.; Pavlovskiy, V.V.; Porsev, V.V.; Evarestov, R.A.; Tunik, S.P. Luminescent or-ganic dyes containing a phenanthro[9,10-*D*]imidazole core and $[Ir(N^\wedge C)(N^\wedge N)]^+$ complexes based on the cyclometalating and diimine ligands of this type. *Dalton Trans.* **2020**, *49*, 6751–6763. https://doi.org/10.1039/d0dt00568a.

[12] Huang, Y.-C.; Li, Z.-B.; Guo, H.-Q.; Mu, D.; Li, H.-Y.; Lu, A.-D.; Li, T.-Y. Synthesis, structures, photophysical properties, and theoretical study of four cationic iridium(III) complexes with electron-withdrawing groups on the neutral ligands. *Inorganica Chim. Acta* **2019**, *496*, 119060. https://doi.org/10.1016/j.ica.2019.119060.
